# Novel Integration of Spatial and Single-Cell Omics Data Sets Enables Deeper Insights into IPF Pathogenesis

**DOI:** 10.3390/proteomes13010003

**Published:** 2025-01-13

**Authors:** Fei Wang, Liang Jin, Xue Wang, Baoliang Cui, Yingli Yang, Lori Duggan, Annette Schwartz Sterman, Sarah M. Lloyd, Lisa A. Hazelwood, Neha Chaudhary, Bhupinder Bawa, Lucy A. Phillips, Yupeng He, Yu Tian

**Affiliations:** 1 Research & Development, AbbVie Bioresearch Center, Worcester, MA 01605, USA; fei.wang1@abbvie.com (F.W.); liang.jin@abbvie.com (L.J.); baoliang.cui@abbvie.com (B.C.); yingli.yang@abbvie.com (Y.Y.); lori.duggan@abbvie.com (L.D.); annette.schwartz@abbvie.com (A.S.S.); 2 Research & Development, AbbVie, South San Francisco, CA 94080, USA; xue.wang@abbvie.com; 3 Research & Development, AbbVie, North Chicago, IL 60064, USAlisa.hazelwood@abbvie.com (L.A.H.); bhupinder.bawa@abbvie.com (B.B.); 4 Research & Development, AbbVie Cambridge Research Center, Cambridge, MA 02139, USA; chaudhary.neha@abbvie.com

**Keywords:** Idiopathic pulmonary fibrosis, spatial transcriptomics, laser capture microdissection, spatial proteomics, mass spectrometry, scRNA-seq, multi-omics integration, cell type, gene signature, protein signature

## Abstract

Idiopathic pulmonary fibrosis (IPF) is a progressive lung disease characterized by repetitive alveolar injuries with excessive deposition of extracellular matrix (ECM) proteins. A crucial need in understanding IPF pathogenesis is identifying cell types associated with histopathological regions, particularly local fibrosis centers known as fibroblast foci. To address this, we integrated published spatial transcriptomics and single-cell RNA sequencing (scRNA-seq) transcriptomics and adopted the Query method and the Overlap method to determine cell type enrichments in histopathological regions. Distinct fibroblast cell types are highly associated with fibroblast foci, and transitional alveolar type 2 and aberrant KRT5-/KRT17+ (KRT: keratin) epithelial cells are associated with morphologically normal alveoli in human IPF lungs. Furthermore, we employed laser capture microdissection-directed mass spectrometry to profile proteins. By comparing with another published similar dataset, common differentially expressed proteins and enriched pathways related to ECM structure organization and collagen processing were identified in fibroblast foci. Importantly, cell type enrichment results from innovative spatial proteomics and scRNA-seq data integration accord with those from spatial transcriptomics and scRNA-seq data integration, supporting the capability and versatility of the entire approach. In summary, we integrated spatial multi-omics with scRNA-seq data to identify disease-associated cell types and potential targets for novel therapies in IPF intervention. The approach can be further applied to other disease areas characterized by spatial heterogeneity.

## 1. Introduction

Idiopathic pulmonary fibrosis (IPF) is an aging-related, progressive and fatal lung disease with an average life expectancy of 3 to 5 years upon diagnosis [1]. IPF has a global prevalence of 10–60 cases per 100,000 people, impacting approximately three million individuals worldwide, with the largest known patient population in East Asia [1]. Therapy options remain limited, and available treatments only delay the disease progression, underscoring the urgent need for a better understanding of IPF pathogenesis to discover novel biomarkers and therapeutic targets [1,2,3]. IPF is characterized by repetitive micro-injuries to local alveolar epithelium, myofibroblast differentiation and excessive deposition of extracellular matrix (ECM) proteins (e.g., collagen) in the surrounding interstitial space, leading to functional decline. Pathologically, IPF lungs exhibit the usual interstitial pneumonia (UIP) pattern with temporal and spatial heterogeneity, where active fibrosis areas with extensive remodeling lie immediately near morphologically normal appearing alveolar tissues [1,4]. The presence of fibroblast foci as local fibrosis centers is a defining pathological hallmark of IPF lungs. These areas are abundant in pathological fibroblast cells as well as collagen fibers and invade adjacent normal alveolar areas to leave behind dense fibrous tissues [5]. These classical histopathological features indicate the high-level spatial and temporal heterogeneity of IPF lungs.

Previous bulk transcriptomics and proteomics studies in human IPF lungs have mainly used whole or regional lung tissues (top versus bottom, central versus peripheral lung regions) to identify gene or protein signatures [6,7,8,9,10,11]. A mass spectrometry-based proteomics study performed compartmental analysis on ECM proteins in distinct anatomical structures, including lung alveolus, airway branch and vasculature [12]. However, spatial heterogeneity and microenvironment complexity pose particular challenges to interpret these bulk analyses. In the past decade, spatial transcriptomics and proteomics technology have successfully captured the whole transcriptome and proteome information from histological or morphological regions of interest (ROIs). For example, GeoMx (NanoString) Digital Spatial Profiler (DSP) and Visium (10x Genomics) platforms generate gene expression data for specific, well-defined ROIs within formalin-fixed paraffin-embedded (FFPE) and fresh frozen tissue sections [13,14]. Laser capture microdissection (LCM) coupled with ultra-sensitive shotgun bottom-up liquid chromatography–mass spectrometry (LC–MS) can quantify spatial protein signatures within a complex tissue biopsy [15]. Deep visual proteomics (DVP) combines artificial intelligence-driven image analysis of cellular phenotypes with automated LCM, further boosting the capacity for molecular profiling [16]. Recently, several spatial transcriptomic and proteomic studies using these cutting-edge techniques have identified differential expression of numerous genes and aberrant activation of multiple signaling pathways by comparing specific pathological regions in IPF lungs [17,18,19,20]. Despite these advancements, the core determinants underlying the initiation and progression of fibrosis from dysregulated cell population cross-talks and cell-matrix interactions remain elusive.

A crucial need in understanding IPF pathogenesis is identifying cell types/clusters associated with specific histopathological regions, particularly fibroblast foci, to understand how cell populations like mesenchymal, epithelial and immune cells as well as ECM components cooperatively compose the fibrotic microenvironment. The ideal data type to address this is spatially resolved single-cell omics. The recent applications of single-cell RNA sequencing (scRNA-seq) approaches have provided significant insights into cellular composition changes with aberrant cell populations and states in human IPF lungs [21,22,23,24]. Remarkable findings include multiple fibroblast cell differentiation, emergence of KRT5-/KRT17+ aberrant epithelial cells, alveolar epithelial cell population shift to airway epithelial cells (e.g., airway basal cells) and SPP1+ macrophages proliferation [21,22,23,24]. However, single cells were originally located in specific niches and spatial information was lost in the cell isolation process. In contrast, the abovementioned GeoMx spatial transcriptomics or LCM-directed LC–MS spatial proteomics expression data are still a mini-bulk blend of all mRNAs or proteins from hundreds of cells in a specific ROI. In addition, deep single-cell proteomics is currently unavailable due to technical limitations. Therefore, if there are only spatial transcriptomics/proteomics data without single cell resolution and scRNA-seq transcriptomics data without spatial information, robust and insightful integration/correlation of spatial versus single-cell data types is required to address the gap.

Currently, there are two major methods: inference-based deconvolution and correlation score-based enrichment, to determine cell type associations in a specific region from omics meta-analysis integrating spatial transcriptomics and scRNA-seq transcriptomics. The inference-based deconvolution method estimates the proportions of each cell type for each region or capture spot, while the correlation-score-based enrichment calculates how strongly a single cellular subtype correlates to a given spatial transcriptomics region [25]. For the protein domain, deep single-cell proteomics is currently unavailable due to technical limitations [26]. Therefore, we lack corresponding single-cell proteomics to integrate with spatial proteomics and must integrate spatial proteomics and scRNA-seq transcriptomics instead. However, traditional methods combining spatial RNA and cellular RNA cannot be applied to integrate spatial protein and cellular RNA due to different feature domains, especially the deconvolution method. To solve this challenge, here we first evaluated two correlation score-based enrichment methods (the Query method and the Overlap method) to integrate spatial RNA and cellular RNA, then innovatively expanded the methods to integrate spatial protein and cellular RNA. These two integrations successfully derived coherent cell type associations in distinct histopathological regions of IPF lungs.

In this study, we first integrated a published GeoMx DSP spatial transcriptomics dataset and a published scRNA-seq transcriptomics dataset in human IPF lungs by adopting the Query method (region-specific gene expression query in the scRNA-seq dataset) and the Overlap method (region-specific gene and cell type-specific gene overlapping) to determine cell type associations and gene signatures in distinct histopathological regions (e.g., fibroblast foci and IPF alveoli). The relatively preserved distant alveolar region in IPF lungs still shows striking cell composition changes, featuring enrichments of transitional alveolar type 2 (AT2) and aberrant KRT5-/KRT17+ epithelial cells. The methods further validated the previous deconvolution method with coherent results. Next, we employed the LCM-directed LC–MS proteomics workflow to detect spatial proteins in fibroblast foci, dense fibrosis and IPF alveoli from IPF lungs with differential and pathway enrichment analysis. By comparing the findings with another published spatial proteomics dataset, common differential expressed protein features and enriched pathways associated with ECM structure organization and collagen processing were highlighted in fibroblast foci. Then, we innovatively reapplied the Query and Overlap methods to integrate spatial proteomics with scRNA-seq transcriptomics and achieved consistent cell type associations in the same histopathological region type compared with spatial transcriptomics and scRNA-seq transcriptomics integration results, corroborating the capability and versatility of the entire approach.

In summary, we integrate spatial multi-omics data with scRNA-seq transcriptomics to identify diseased cell types and representative signatures in distinct histopathological regions in human IPF lungs, providing valuable insights for future mechanistic studies in IPF pathogenesis. The methods can be readily reapplied to investigate other disease areas characterized by spatial heterogeneity, such as cancer and autoimmune diseases.

## 2. Materials and Methods

### 2.1. Reanalysis of Published Omics Datasets Used in the Integration and Comparison

#### 2.1.1. Reanalysis of the Eyres Spatial Transcriptomics Dataset

Preprocessed mRNA count data after filtering and Q3/upper quartile normalization from NanoString GeoMx DSP spatial transcriptomics in human IPF lungs were directly downloaded from Table S1 in Eyres et al. *Cell Reports* 2022 [17] and further processed using R to derive region-specific differential gene sets. We referred to it as the Eyres spatial transcriptomics dataset thereafter (Table 1). A total of 1085 genes, including 75 ECM genes annotated with matrisome genes [27], were identified within seven histopathological region types: control alveoli (n = 12) and control blood vessel (n = 6) from normal control donors (n = 3), as well as IPF adjacent alveoli (n = 10), IPF blood vessel (n = 6), IPF distant alveoli (n = 10), IPF fibroblastic foci (n = 10) and IPF immune infiltrate (n = 6) from IPF patients (n = 3) (Supplementary Table S1).

A non-parametric Wilcoxon rank-sum test (“wilcox.test” function from “stats” R package, version 4.2.2) was used to identify differentially expressed genes between two histopathological region types of interest. Based on the original paper [17], genes with a Log2 fold change (FC) of 0.25 and a Benjamini–Hochberg-adjusted *p*-value or false discovery rate (FDR) less than 0.05 were considered differentially expressed with statistical significance.

#### 2.1.2. Reanalysis of the Herrera Spatial Proteomics Dataset

Raw MaxQuant proteomics intensity file from a published LCM-directed LC–MS spatial proteomics dataset (PXD029341) in human IPF lungs from Herrera et al. JCI insight 2022 [19] was downloaded and further processed using R. We referred to it as the Herrera spatial proteomics dataset thereafter (Table 1). The protein abundance data was filtered to ensure that each protein group contained no more than 50% missing values in all sample groups or no more than 30% missing values in any region type. The protein abundance data were then Log2 transformed and normalized using the “normalizeCyclicLoess” function in the “limma” R package (version 3.50.0) [29]. The remaining missing values were imputed using the “missForest” function in the “missForest” R package (version 1.4) [30]. A total of 2835 protein IDs, including 176 ECM proteins annotated with matrisome proteins [27], were identified within four histological region types: control alveoli (n = 6) from normal control donors (n = 6), as well as IPF alveoli (n = 6), IPF fibrosis (n = 6) and IPF fibroblastic foci (n = 6) from UIP/IPF patients (n = 6) (Supplementary Table S1).

Differential expression analysis was performed using the “limma” R package (version 3.50.0) [29,31]. Proteins with an FC of 1.5 and a Benjamini–Hochberg-adjusted *p*-value or FDR less than 0.05 were considered differentially expressed with statistical significance.

#### 2.1.3. Comparisons of the Eyres Spatial Transcriptomics Dataset and the Herrera Spatial Proteomics Dataset

There are three common histopathological region types between the Eyres spatial transcriptomics dataset and the Herrera spatial proteomics dataset: IPF fibroblast foci, IPF adjacent alveoli and control alveoli. We performed three pairwise comparisons: IPF fibroblast foci versus IPF adjacent alveoli; IPF fibroblast foci versus control alveoli; IPF adjacent alveoli versus control alveoli in 320 common features between genes from spatial transcriptomics and proteins from spatial proteomics (Supplementary Figure S1A,B). Common differential expressed genes and proteins with FDR < 0.05 were highlighted in the scatter plots (Supplementary Figure S1C–E).

In addition, the enriched pathways in up-regulated differentially expressed genes (Log2 FC > 0.25, FDR < 0.05) or proteins (FC > 1.5, FDR < 0.05) comparing fibroblast foci versus IPF adjacent alveoli were determined by over-representation analysis using the 2019 WebGestalt interface: https://2019.webgestalt.org/ (accessed on 15 June 2024) against the non-redundant Gene Ontology terms of Biological Process [32]. 

### 2.2. Integration of GeoMx Spatial Transcriptomics and scRNA-Seq Transcriptomics

#### 2.2.1. Extraction of Region-Specific Differentially Expressed Genes from the Eyres Spatial Transcriptomics Dataset

There are seven histopathological region types from the Eyres spatial transcriptomics dataset. The principal component analysis was performed using the “prcomp” function in R. A non-parametric Wilcoxon rank-sum test (“wilcox.test” function from “stats” R package, version 4.2.2) was used to identify differentially expressed genes between the target histopathological region type and all other six non-target types. A Log2 FC > 0.25 and FDR < 0.05 were used to identify up-regulated differentially expressed genes, which are signature genes representing the respective region types. Based on the criteria, there are no region-specific gene signatures for IPF adjacent alveoli and control blood vessel. This method extracted 32 up-regulated region-specific differential genes for control alveoli, 27 for IPF blood vessel, 41 for IPF distant alveoli, 50 for IPF fibroblast foci and 106 for IPF immune infiltrate. Both the following Query and Overlap integration methods require these up-regulated region-specific differential genes as a starting point.

#### 2.2.2. Query Method: Query of Region-Specific Differential Gene in Distinct Cell Types to Compute Z-Scores Based on Relative Expression Levels

The workflow of the Query method is shown in Supplementary Figure S2A in detail. Raw counts with Seurat object [33] from a published human IPF lung scRNA-seq dataset (GSE135893) in Habermann et al. Science Advances 2020 [21] offered 33,694 gene expression within 89,326 cells (31,644 cells from 10 normal control donors and 57,682 cells from 12 IPF patients) and processed further using “Seurat” 5.0.1, “Matrix” 1.6-0 and “tidyr” 1.3.0 R packages. We referred to it as the Habermann scRNA-seq transcriptomics dataset thereafter (Table 1). After filtering out genes expressed in less than 10 cells (keep_genes method in “Seurat”), 24,470 genes remained. Notably, 95.6% of genes (1037 out of 1085) from the Eyres spatial transcriptomics dataset are also listed in these 24,470 genes. mRNA raw counts were then normalized and scaled using the LogNormalize method (“NormalizeData” function in “Seurat”) in each cell [33].

There are five large cell types (Epithelium, Mesenchyme, Myeloid, Endothelium and Lymphoid), which can be further divided into 30 cell types, either from normal control donors or IPF patients, within these 89,326 cells. A total of 180 HAS1+ high fibroblast cells only appeared in one specific IPF patient. Thus, this cell type was removed from the following analysis.

Normalized mRNA count for gene **i** in cell **j**, or **X_ij_**, is firstly transformed as:

**Y_ij_** = (**X_ij_** − **μ_i_**)/**σ_i_**

where **μ_i_** is the average expression level of gene **i** among all 89,326 cells and **σ_i_** is the standard deviation of gene **i** expression level among all 89,326 cells.

**Figure 1:**
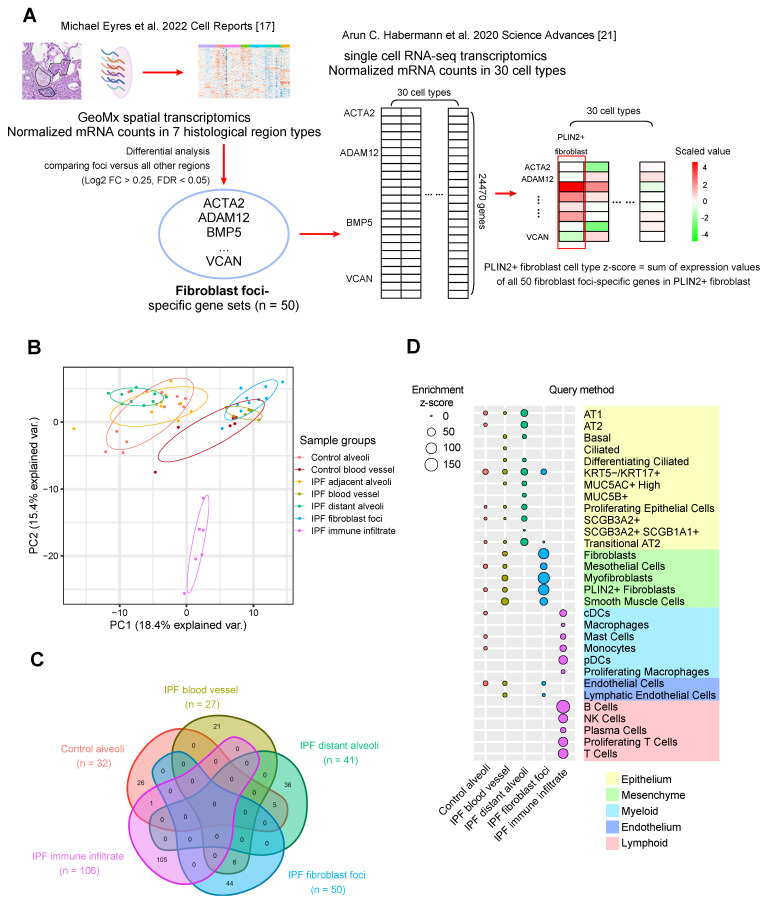
Cell type enrichments in distinct region types integrating GeoMx spatial transcriptomics and scRNA-seq transcriptomics in human IPF lungs by the Query method. (**A**) Workflow of the Query method. In this example, differential analysis extracted up-regulated fibroblast foci-specific genes (n = 50) from GeoMx spatial transcriptomics [17], and the sum expression of the whole gene set was queried as a z-score in PLIN2+ fibroblast cells from scRNA-seq transcriptomics [21]. (**B**) PCA plotting of gene expression pattern. (**C**) Venn graph of the up-regulated region-specific gene sets in five histopathological region types: 32 up-regulated region-specific differential genes for control alveoli, 27 for IPF blood vessel, 41 for IPF distant alveoli, 50 for IPF fibroblast foci and 106 for IPF immune infiltrate. (**D**) Enrichment z-score summary of 30 cell types in five histopathological region types from spatial transcriptomics. These 30 cell types are classified into five large types: Epithelium, Mesenchyme, Myeloid, Endothelium and Lymphoid. Abbreviations. ACTA2: Smooth muscle alpha-actin; ADAM12: Disintegrin and metalloproteinase domain-containing protein 12; BMP5: Bone morphogenetic protein 5; VCAN: Versican; PC: principal component; AT1: Alveoli type 1 epithelial cells; AT2: Alveoli type 2 epithelial cells; KRT5: Keratin 5; KRT17: Keratin 17; MUC5AC: Mucin 5AC; MUC5B: Mucin 5B; SCGB3A2: secretoglobin family 3A member 2; SCGB3A1: secretoglobin family 3A member 1; PLIN2+: perilipin 2; cDCs: Conventional dendritic cells; pDCs: Plasmacytoid dendritic cells; NK cells: Natural killer cells. Cell type annotations from all figures follow the same abbreviations.

The cellular average expression of gene **i**, or **Z_i_**, in a cell type is defined by summing all normalized mRNA count **Y_ij_** from all cells in this cell type, then dividing by cell number in this cell type. Then **Z_i_** of all up-regulated region-specific differential genes was summed to obtain the z-score of a specific region in this cell type (Figure 1A). For regions from IPF patients, including IPF blood vessel, IPF distant alveoli, IPF fibroblast foci and IPF immune infiltrate, single cells from IPF patients were used to calculate the z-score in each cell type. For control alveoli region from normal control donors, single cells from control donors were used to calculate the z-score in each cell type.

The Query method was referred from Huang et al. 2023 BMJ Open Respiratory [11]. If one gene is highly expressed in one pathological region and one cell type is specifically distributed in this region, this cell type should also express a relatively high level of this gene. Summarized expression z-scores for each region in each cell type were listed in Figure 1D (enrichment) and Supplementary Figure S3A (depletion). Cell type enrichment and depletion were ranked as cell type/region associations.

#### 2.2.3. Overlap Method: Use the Degree of Overlap Between Region- and Cell Type-Specific Differential Genes to Calculate Cell Type Enrichments

The workflow of the Query method is shown in Figure 1A generally and in Supplementary Figure S2B in detail. An unpaired two-tailed Student’s *t*-test (*p* < 10^−5^, calculate effect size with Cohen’s d value, using “rstatix” 0.7.2 R package) was used to identify differentially expressed genes between the target IPF cell type of interest and all other non-target IPF cell types. The same method was applied to identify differentially expressed genes between the target control donor cell type of interest and all other non-target control donor cell types. For each gene with *p* < 10^−5^, we examined for which cell type the effect size was highest and determined that gene to be specific or uniquely up-regulated for this cell type. Consequently, there are no overlapping cell type-specific genes from different cell types, thereby reducing interferences from confounding scoring [25].

**Figure 2:**
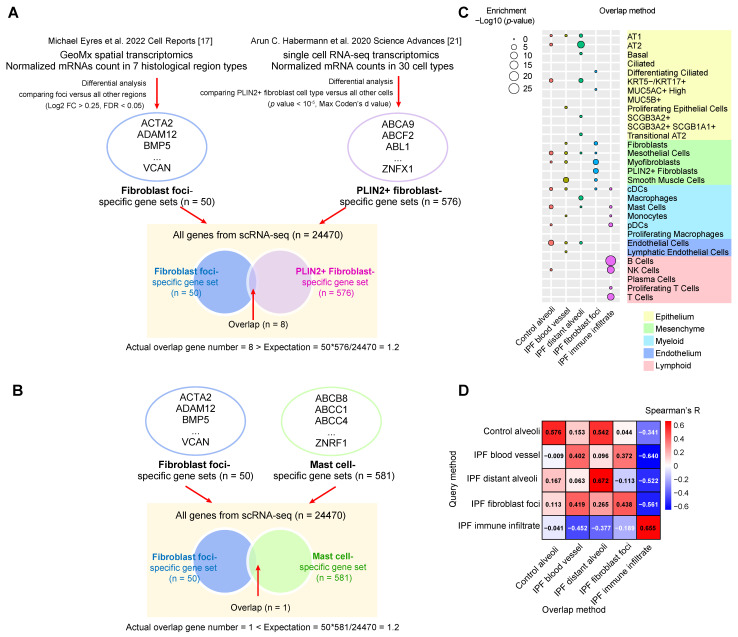
Cell type enrichments in distinct region types integrating GeoMx spatial transcriptomics [17] and scRNA-seq transcriptomics [21] in human IPF lungs by the Overlap method. (**A**,**B**) Two representative workflow examples of the Overlap method. (**A**) Enrichment example: Differential analysis extracted up-regulated fibroblast foci-specific genes (n = 50) from GeoMx spatial transcriptomics and PLIN2+ fibroblast-specific genes (n = 576) from scRNA-seq transcriptomics and determined their overlap is larger than expected by random, indicative of enrichment. (**B**) Depletion example: Differential analysis extracted up-regulated fibroblast foci-specific genes (n = 50) from GeoMx spatial transcriptomics and mast cell-specific genes (n = 581) from scRNA-seq transcriptomics and determined their overlap is smaller than expected by random, indicative of depletion. (**C**) Enrichment *p*-value summary of 30 cell types in five histopathological region types from spatial transcriptomics. (**D**) Spearman’s correlations between cell type rankings in five histopathological defined region types from the Query method and the Overlap method. Abbreviations. ACTA2: Smooth muscle alpha-actin; ADAM12: Disintegrin and metalloproteinase domain-containing protein 12; BMP5: Bone morphogenetic protein 5; VCAN: Versican; ABCA9: ATP binding cassette subfamily A member 9; ABCF2: ATP binding cassette subfamily F member 2; ABL1: ABL proto-oncogene 1; ZNFX1: Zinc finger NFX1-type containing 1; ABCB8: ATP binding cassette subfamily B member 8; ABCC1: ATP binding cassette subfamily C member 1; ABCC4: ATP binding cassette subfamily C member 4; ZNRF1: Zinc and ring finger 1.

Referred from a pancreatic ductal adenocarcinoma omics integration paper [34], we calculated the significance of the overlap items between up-regulated IPF region-specific and IPF cell type-specific differentially expressed genes, as well as control donor regions-specific and control donor cell type-specific differentially expressed genes, using the hypergeometric cumulative distribution with “phyper” function in “stats” 4.2.2 R package. All 24,470 genes from the Habermann scRNA-seq transcriptomics dataset worked as the background to compute *p*-values and determine whether their overlap is higher (enrichment) than expected by chance. In parallel, we tested for cell type depletion by computing −Log10 (1 − *p*) and determined whether their overlap is lower (depletion) than expected. The significance *p*-values were listed for each region in each cell type in Figure 2C (enrichment) and Supplementary Figure S3B (depletion).

Finally, we listed z-scores from the Query method and significance *p*-values from the Overlap method for all cell types in the same region type and computed Spearman’s R coefficients to test the positive correlations.

#### 2.2.4. Comparisons Between Deconvolution Methods and Enrichment Methods

To directly compare the inference-based deconvolution method with the Query and the Overlap enrichment methods, we used the NanoString-introduced SpatialDecon R package [35] to deconvolute the relative cell type proportions in each ROI from the Eyres spatial transcriptomics dataset based on the Habermann scRNA-seq transcriptomics dataset reference and computed the mean cell type proportions for each specific region type.

### 2.3. LCM-Directed LC–MS

For LCM-directed LC–MS proteomics data analysis and integration, we primarily relied on the Herrera spatial proteomics dataset [19]. However, we also performed confirmatory experiments in-house for this study. The following method describes the in-house experimental approach.

#### 2.3.1. LCM Sample Preparations

FFPE blocks of IPF lung tissues were sourced from Folio (n = 1, Subject ID: D9160, Sample ID: F14739.C2d) and Discovery Life Biosciences (n = 1, D25139, Sample ID: F33840.5B). On Day 1, six serial sections, each 5 μm thick, were cut per block. The first section was mounted on a regular glass slide (Surgipath Apex Superior adhesive slides, Leica: #3800080) and stained with hematoxylin and eosin (Sigma: GHS1128-4L). The next five serial sections were mounted on Pen Membrane slides (Leica: #11505189) and stained with Meyer’s hematoxylin for 30 s, then held at 4 °C until Day 3. The hematoxylin and eosin (H&E) slides were digitally imaged using a Panoramic 250 whole slide scanner at 20× magnification (3DHISTECH, Budapest, Hungary).

On Day 2, the images were imported into Visiopharm software (version 2020.01), and ROIs were annotated by a pathologist. Histopathological region types included fibroblast foci, chronic fibrosis, and adjacent near-normal alveoli. On Day 3, the annotated H&E images were used to guide the manual LCM capture on the Mayers hematoxylin-stained Pen membrane slides. For each ROI, 5 mm^2^ of tissue area was captured into Eppendorf caps and stored at −80 °C for subsequent sample preparations.

#### 2.3.2. LC-MS

Proteins from LCM blocks were extracted with 5 µL lysis buffer (6 M guanidinium chloride, 10 mM tris(2-carboxyethyl)phosphine, 20 mM iodoacetamide, 100 mM Tris, pH 8.5) at 95 °C for 60 min. Samples were spun down every 15 min during heating and then sonicated for 20 min with a water bath. The lysate was diluted at a ratio of 1:10 with dilution buffer containing LysC/trypsin (1:10, enzyme: protein), followed by overnight digestion at 37 °C. To terminate the digestion and acidify samples, 50 µL of 2% trifluoroacetic acid was added. After spinning down, the supernatant was transferred into SDB-RPS tips (CDS Analytical, Oxford, PA, USA). The tip was centrifuged at 6000 rpm for 5 min for complete flow-through. The loading process was repeated once more. The tip was washed twice with 100 µL 0.2% trifluoroacetic acid. The peptides were eluted twice with 50 µL elution buffer containing 5% ammonium hydroxide and 80% acetonitrile. The dried peptides were subsequently resuspended with LC injection buffer (2% methanol, 0.1% trifluoroacetic acid in water).

Peptide samples were analyzed using a Thermo Scientific EASY-nLC 1000 system coupled to an Orbitrap Fusion Lumos Mass Spectrometer (Thermo Scientific, Waltham, MA, USA). The LC system was equipped with a PepMap™ RSLC C18 column (75 µm × 75 cm, 2 µm, Thermo Scientific, Waltham, MA, USA) maintained at 50 °C. Solvent A consisted of 0.1% formic acid in water, and solvent B consisted of 0.1% formic acid in 90% acetonitrile. A 400 ng peptide sample was loaded onto a trap with 5% acetonitrile at 10 μL/min flow rate for 5 min and then analyzed at 200 nL/min with the following LC separation gradient: 5–15% B for 5 min, 15–30% B for 170 min, 30–65% B for 20 min, 65–95% B for 11 min. Data collection was operated in a 3 s cycle using the data-dependent top-speed mode with high-field asymmetric waveform ion mobility spectrometry (FAIMS). The scanning event occurred at 4 stepping compensation voltages: −45 V, −60 V, −75 V and –90 V. The MS1 survey scan (*m*/*z* 400-1500) was at a resolution of 120,000 (FWHM @ *m*/*z* = 200), with an automated gain control target of 400,000 and a maximum injection time of 50 ms. Precursors were fragmented in high-energy collisional dissociation activation mode at a normalized collision energy of 30%, and the dynamic exclusion was set at 45 s. Precursors were filtered by quadrupole using an isolation window of 1.2 atomic mass units. The MS2 spectra were collected at a resolution of 15,000 in the ion trap, with an automated gain control target of 10,000 and a maximum injection time of 35 ms.

#### 2.3.3. Proteomics Data Analysis

A database search of mass spectrometry raw files was performed using FragPipe (version 1.8) [36] against a Uniprot human protein database (downloaded on 12 January 2021). Default settings were used for MSFragger (version 3.5), and MS1 quantification was enabled with the maxLFQ option selected. The following analysis was performed in the R framework (version 4.1.2) and was the same as the reanalysis pipeline for the Herrera spatial proteomics dataset.

Differential expression analysis was performed using the “limma” R package (version 3.50.0) [29,31]. Proteins with an FC of 1.5 and a nominal *p*-value less than 0.05 were considered differentially expressed with statistical significance. The enriched pathways in up-regulated differentially expressed proteins comparing fibroblast foci versus IPF alveoli were determined by over-representation analysis using the 2019 WebGestalt interface: https://2019.webgestalt.org/ (accessed on 15 June 2024) against the non-redundant Gene Ontology terms of Biological Process [32]. 

### 2.4. Integration of LCM-Directed LC–MS Spatial Proteomics and scRNA-Seq Transcriptomics

There are four histopathological region types from the Herrera spatial proteomics dataset. An unpaired two-tailed Student’s *t*-test (*p* < 10^−2^, calculate effect size with Cohen’s d value) was used to identify differentially expressed proteins between the target region type and all other three non-target types. For each gene with *p* < 10^−2^, we examined for which region the effect size was highest and determined that protein to be specific or uniquely up-regulated for this region type. Notably, 95.0% of corresponding genes from the Herrera spatial proteomics dataset (2694 out of 2835) are also listed in the 24,470 genes from the Habermann scRNA-seq transcriptomics dataset. We extracted 407 up-regulated region-specific differential proteins for control alveoli, 77 for IPF alveoli, 189 for IPF fibrosis, and 172 for IPF fibroblastic foci. Next, we integrated these protein lists with the Habermann scRNA-seq transcriptomics dataset by querying z-scores of corresponding genes from up-regulated region-specific differential proteins in distinct cell types and by calculating the significance of overlap items between up-regulated region-specific differential proteins and cell type-specific differential genes.

## 3. Results

ECM proteins play a critical role in IPF pathogenesis [4]. The ideal method to study IPF pathogenesis, involving driver cell types and key protein signatures featuring ECM in pathological regions, would utilize spatially resolved single-cell proteomics data. However, due to technical constraints, we need to take a stepwise approach using available technologies and datasets. The most suitable approach is to integrate LCM-directed LC–MS spatial proteomics with corresponding scRNA-seq transcriptomics. To the best of our knowledge, there is limited precedence for this method. The current study presents our attempt to tackle this issue. The workflows are: 1. Selection of spatial omics data to integrate with scRNA-seq transcriptomics data. 2. Development of an appropriate method for spatial proteomics and scRNA-seq transcriptomics data integration based on the evaluation of spatial transcriptomics and scRNA-seq data integration methods. 3. Result comparisons of integrated spatial transcriptomics and scRNA-seq transcriptomics versus spatial proteomics and scRNA-seq transcriptomics, alongside new insights for IPF pathogenesis.

### 3.1. Comparisons of Spatial Transcriptomics and Spatial Proteomics in the Common Histopathological Region Types

The ideal multi-omics data of spatial transcriptomics and spatial proteomics for the following integration require tissue slices from the same patient tissue block and the same histopathological area selections. Due to the absence of such datasets, two independent studies were selected: a published NanoString GeoMx DSP spatial transcriptomics IPF dataset [17] (referred to as Eyres) and a published LCM-directed LC–MS spatial proteomics IPF dataset [19] (referred to as Herrera) (Table 1). To confirm the suitability of these two datasets, we performed comparisons using key differential expression features and key pathways as comparison endpoints. Their feature expression matrices were extracted and reanalyzed through data clean-up, differential and pathway enrichment analysis workflow (See methods for details). The Eyres spatial transcriptomics dataset identified a total of 1085 gene IDs within seven histopathological region types: control alveoli and control blood vessel from normal control donors; IPF blood vessel, IPF adjacent alveoli, IPF distant alveoli, IPF fibroblastic foci and IPF immune infiltrate from IPF patients (n = 3). The Herrera spatial proteomics dataset identified a total of 2835 protein IDs within four histopathological region types: control alveoli from normal control donors (n = 6); IPF fibroblast foci, IPF adjacent mature fibrosis, and IPF adjacent alveoli from IPF/UIP patients (n = 6).

A comparison of common features from two datasets identified 320 overlapping total feature IDs and 36 overlapping ECM feature IDs (Supplementary Figure S1A). Then, three common histopathological region types between two datasets will be the main target regions: IPF fibroblast foci and IPF (adjacent) alveoli and control alveoli. Expression folds change heatmaps in three pairwise region-region comparisons among 320 overlapping features in either transcriptome or proteome display robust positive correlations (Supplementary Figure S1B). This confirms the capacity of spatial omics to delineate feature expression patterns in histologically defined regions. Among the 320 common detected features, 20 features are both up-regulated, and 13 features are both down-regulated in fibroblast foci compared with IPF alveoli (both FDR < 0.05 considered significant, Supplementary Figure S1C). 24 features are both up-regulated, and 29 features are both down-regulated in fibroblast foci compared with control alveoli (Supplementary Figure S1D). One feature (COL3A1) is up-regulated, and one feature (GBP1) is down-regulated in IPF alveoli compared with control alveoli (Supplementary Figure S1E).

Moreover, enriched biological processes were analyzed in terms of Gene Ontology (GO) using over-representation analysis (ORA) on either up-regulated differentially expressed (DE) genes or proteins when comparing fibroblast foci versus IPF alveoli region type. Both results highlighted pathways including collagen metabolic process, extracellular structure organization and bone development (Supplementary Figure S1F,G and Supplementary Tables S2 and S3), underscoring fibroblast foci as local fibrogenesis sites.

Taken together, all these data suggest that selected spatial omics studies can accurately detect feature expression in histologically defined areas with cross-validation. This provides the foundation for combining either spatial transcriptomics or proteomics data with single-cell transcriptomics data to deduce cell type enrichments based on DE features in the following integration.

### 3.2. Integration of Spatial Transcriptomics and scRNA-Seq Transcriptomics to Deduce Cell Type Enrichments in Pathological Regions

To illustrate the integration methods and identify driver cell types/clusters and signature genes in specific pathological regions in human IPF lungs, the Eyres spatial transcriptomics dataset was integrated with a published scRNA-seq transcriptomics IPF dataset [21] (referred to as Habermann) using two correlation score-based enrichment methods: the Query method and the Overlap method.

#### 3.2.1. Query Method

The workflow of the Query method is shown in Figure 1A generally and in Supplementary Figure S2A in detail, as referred from an IPF regional transcriptomics study [11]. This method involves querying the relative expression level of up-regulated region-specific differential genes in distinct cell types within the scRNA-seq transcriptomics dataset, hence the name Query method. For example, if a fibroblast foci-derived gene set is highly expressed in a particular cell type, it suggests that this cell type is enriched in the fibroblast foci area.

The first step is to extract up-regulated region-specific genes by differential analysis. As previously introduced, the Eyres spatial transcriptomics dataset provided spatial gene expression within seven histopathological region types: control alveoli, control blood vessel, IPF blood vessel, IPF adjacent alveoli, IPF distant alveoli, IPF fibroblastic foci and IPF immune infiltrate (Figure 1B). Based on the original paper [17], an adjusted *p*-value or FDR cut-off of 0.05 and a Log2 FC cut-off of 0.25 by Wilcoxon rank-sum test were used to identify up-regulated DE genes between the target region types and all other six non-target types. These gene sets are defined as signatures representing their respective regions (Figure 1C and Supplementary Table S4). Based on the cut-off criteria, these region-specific gene sets are almost exclusive, without overlapping genes (Figure 1C), thereby preventing confounding scoring from overlapping gene signatures [25,34]. Notably, there are no specific gene signatures for control blood vessel and IPF adjacent alveoli regions under the cut-off criteria. From the PCA (Principal Component Analysis) plot on the expression pattern (Figure 1B), the two regions have large intra-group variations and overlap with other regions, which makes it difficult to discern specific up-regulated DE genes with prominence. Consequently, the following integration only focuses on the remaining five region types.

The IPF fibroblast foci-specific gene set (n = 50) was used as an example to demonstrate the Query method (Figure 1A). The Habermann scRNA-seq transcriptomics dataset provided 24,470 gene expression with 89,326 isolated cells, classifying into 30 annotated cell types from control donor and IPF lungs (See methods for details). The average relative expression level of 24,470 genes in each cell type was calculated, followed by querying and summing the expression levels of all 50 IPF fibroblast foci-specific genes in each cell type as z-scores (Figure 1A and Supplementary Table S5). Similarly, z-scores were calculated for each cell type in each of the five region types. A positive z-score means enriched expression of up-regulated region-specific genes in a cell type (Figure 1D), whereas a negative z-score means depleted expression (Supplementary Figure S3A). The absolute value represents the overall extent of enrichment or depletion. For clear visualization, the enrichment (Figure 1D) and depletion matrix maps (Supplementary Figure S3A) are separated.

The cell type enrichment z-scores were calculated in control alveoli from normal donors and IPF blood vessel, IPF distant alveoli, IPF fibroblast foci and IPF immune infiltrate from IPF patients (Figure 1D). Control alveoli region exhibits enrichment with multiple epithelial, endothelial, mesenchymal, and myeloid cell types. IPF blood vessel region is enriched with smooth muscle cells and epithelial and endothelial cells. IPF fibroblast foci region is enriched with multiple mesenchymal cell types like fibroblasts, PLIN2+ fibroblasts and myofibroblasts, while IPF immune infiltrate region is enriched exclusively with diverse immune cell types including B cells, T cells, NK cells, monocytes, and macrophages (Figure 1D).

When comparing morphologically normal distant alveoli (areas far from fibrosis sites) from IPF patients to control alveoli from normal donors, IPF alveoli regions show remarkable enrichments in epithelial cell types and depletions in mesothelial and immune cell types. The enrichment of AT1, AT2 and transitional AT2 in IPF alveoli regions suggests active tissue repair with epithelial renewal [17,37,38]. In addition, the pathological KRT5-/KRT17+ aberrant basaloid epithelial cell population, which has been independently identified in multiple studies [21,22,39,40], is enriched in IPF alveoli region as well as fibroblast foci regions. These cells lie surrounding the fibroblast foci area, express markers of epithelial-mesenchymal transition and senescence-related genes and persist as an intermediate, non-regenerative state [22]. Based on the recent cell trajectory analysis, AT2 to transitional AT2 lineage branches to either regenerative AT1 or non-regenerative KRT5-/KRT17+ epithelial cells [20]. The enrichments of KRT5-/KRT17+ and transitional AT2 cells in the distant alveoli region may predict the dysregulated homeostasis in the future fibrosis progression. These findings reveal significant spatially discrete cell type enrichment changes between IPF and control donor region types. Even the morphologically normal regions of fibrotic lungs exhibit considerable cellular and molecular dysfunction and architectural remodeling.

#### 3.2.2. Overlap Method

Next, we adopted another integration method referred from a pancreatic ductal adenosarcoma paper [34]. The approach extracts up-regulated region-specific genes and cell type-specific genes and determines their overlap significance, which is termed the Overlap method (Figure 2A and Supplementary Figure S2B). For example, if a certain cell type is enriched in a particular region, it should have overlapping specific gene sets, similar to the ORA method in the pathway enrichment analysis [41].

An unpaired two-tailed Student’s *t*-test (*p* < 10^−5^, calculate effect size with maximum Cohen’s d value) was used to identify cell type-specific genes between the target cell type of interest and all other cell types (Supplementary Tables S6 and S7) [25,34]. The significance of overlap items between up-regulated region-specific and cell type-specific genes was then calculated to determine whether their overlap is higher (cell type enrichment, Figure 2A) or lower (cell type depletion, Figure 2B) than expected by chance. For instance, fibroblast foci-specific genes significantly overlap with PLIN2+ fibroblast-specific genes (Figure 2A), in contrast to mast cell-specific genes (Figure 2B). Extending this analysis to all pairs of regions and cell types produced an association matrix (Supplementary Table S8). The enrichment matrix (Figure 2C) and depletion matrix (Supplementary Figure S3B) are separated for clear visualization. Furthermore, the overlapping up-regulated region-specific (Supplementary Table S4) and cell type-specific genes (Supplementary Tables S6 and S7) in all region/cell type pairs are listed in Supplementary Tables S9–S13.

Compared with the Query method, the Overlap method requires a higher threshold to show positive associations between cell types and regions, resulting in fewer enriched cell types in a specific region type. However, major cell types enriched in each region from the two methods are quite consistent: control alveoli region is enriched with diverse large cell types. IPF immune infiltrate region is enriched with immune cell types. IPF fibroblast foci region is enriched with multiple mesenchymal cell types like fibroblasts, PLIN2+ fibroblasts and myofibroblasts. IPF blood vessel region is enriched with smooth muscle cells. AT1, AT2, transitional AT2 as well as KRT5-/KRT17+ epithelium cells are enriched in the IPF distant alveoli region. In sum, 30 cell type enrichment z-scores from the Query method and significance *p*-values from the Overlap method in each of the five region types are ranked together, and they display strong positive correlations in corresponding region types, cross-validating the integration results (Figure 2D).

To further confirm the two enrichment methods, the Query and Overlap results were compared with the inference-based deconvolution result. The relative cell type proportions in each region from the Eyres spatial transcriptomics dataset were deconvoluted from the same Habermann scRNA-seq transcriptomics dataset employed in the current study. Additionally, mean cell type proportions in histopathological region types were directly obtained from another human IPF lung GeoMx spatial transcriptomics dataset in Blumhagen et al. 2023 Respiratory Research [18] (Table 1). They performed the deconvolution based on another independent scRNA-seq transcriptomics dataset reference [22] (Table 1). Distinct cell number proportions in histopathological region types were also extracted from a new BioRixiv human IPF lung Xenium in situ dataset [28] (Table 1). Currently, the Xenium platform is a novel, high-resolution, imaging-based spatial profiling technology detecting RNA targets in pre-designed gene panels (~400 genes) with single-cell resolution in the tissue context [42]. Table 1 summarizes the comprehensive background information in these datasets, and Supplementary Table S14 summarizes the cell type enrichment rankings from the Query and Overlap methods, cell type proportion estimates from the deconvolution method and cell number proportions from the Xenium measurement in distinct histopathological region types. It should be noted that detailed cell type annotations are not identical in these comparisons. Some histopathological region types are unique to one specific dataset and absent in other datasets (e.g., immune infiltrate region type only appears in the Eyres spatial transcriptomics dataset). These orthogonal and complementary methods, either from computation-based integration (query/overlap/deconvolution) or experiment-based cell number count (Xenium), generate consistent cell type ranking to attain cell type composition changes in pathological regions during disease progression. For example, the top three abundant cell types identified from the Eyres dataset deconvolution method are also top-ranked in other ranking results (see Supplementary Table S14 with color highlighting). In summary, the integration results indicate spatially discrete distributions of cell types in IPF lungs and highlight the pathogenesis mechanisms, especially in morphologically normal alveolar regions as well as fibroblast foci regions in IPF lungs.

### 3.3. LCM-Directed LC–MS Proteomics Identified Key ECM Proteins and Related Pathways from Human IPF Lung Tissues

After evaluating whether the Query method and the Overlap method can integrate spatial/single-cell transcriptomics to obtain consistent cell type/region associations with computation-based deconvolution and imaging-based cell counting, it is natural to assess whether current principles can be expanded and innovatively integrate spatial proteomics with single-cell transcriptomics. This is particularly intriguing given that single-cell proteomics data is not feasible.

To prepare for this, spatial protein profiling is required to extract up-regulated region-specific protein features in histologically defined areas, such as fibroblast foci. To this end, distinct pathological regions (fibroblast foci: n = 2, fibrosis: n = 3, IPF alveoli: n = 2, each with 5 mm^2^ tissue area) from two commercial human IPF lung FFPE blocks were subjected to the in-house LCM-directed LC–MS-based quantitative proteomics pipeline (Supplementary Figure S4A,B). This analysis identified a total of 2888 proteins, including 170 ECM proteins (88 core matrisome proteins and 82 matrisome-associated proteins) based on matrisome annotations [27] (Supplementary Figure S4C). Differential analysis comparing fibroblast foci versus IPF alveoli region identified 88 up-regulated and 120 down-regulated DE proteins (|FC| > 1.5, nominal *p*-value < 0.05), including 17 up-regulated and 30 down-regulated DE ECM proteins (Supplementary Figure S4D and Supplementary Table S15). Comparison of fibrosis versus IPF alveoli region identified 12 up-regulated and 13 down-regulated DE total proteins, including two up-regulated and one down-regulated DE ECM proteins (Supplementary Figure S4E and Supplementary Table S16). A comparison of fibroblast foci versus fibrosis region identified 35 up-regulated and 116 down-regulated DE total proteins, including 11 up-regulated and 26 down-regulated DE ECM proteins (Supplementary Figure S4F and Supplementary Table S17).

Next, the in-house protein profiling data was compared to the Herrera spatial proteomics data [19]. As previously introduced, they interrogated fibroblast foci, fibrosis, and IPF alveoli in six IPF/UIP patients and control alveoli in six normal control donors using LCM-directed LC–MS. They identified 2835 total and 176 ECM proteins, with approximately 80% overlapping with in-house results (Supplementary Figure S4G). Further differential analysis revealed 23 commonly up-regulated (e.g., TNC, PLOD1, FKBP10, P4HA1, COL5A1 and VCAN) and 18 down-regulated (e.g., ANXA3, COL6A6, PRX and MMP9) DE proteins when comparing fibroblast foci versus IPF alveoli (Supplementary Figure S4H). Similarly, we analyzed the enriched biological processes using ORA (Supplementary Table S18). The up-regulated DE proteins comparing fibroblast foci versus IPF alveoli primarily represented pathways like protein hydroxylation, collagen metabolic process, peptidyl-proline modification, and extracellular structure organization, which are consistent with the Herrera results (comparing Supplementary Figures S1G and S4I). Together, the in-house spatial proteomics data are cross-validated with the published Herrera data and suggest active fibrous collagen biosynthesis in the fibroblast foci, thereby confirming the ability of LCM-directed LC–MS spatial profiling to extract protein features in histopathological regions. Given that the in-house dataset lacks normal donor controls and the Herrera dataset has a larger sample size, this published dataset will be used for the following integration analysis.

### 3.4. Integration of Spatial Proteomics and scRNA-Seq Transcriptomics by the Query Method and the Overlap Method

**Figure 3:**
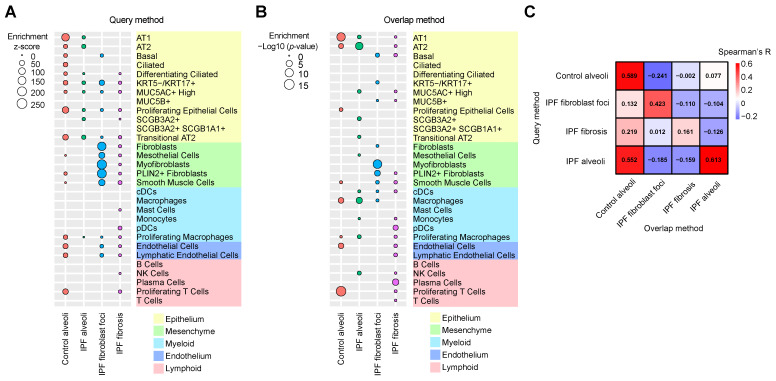
Cell type enrichments in distinct region types integrating LCM-directed LC–MS spatial proteomics and scRNA-seq transcriptomics in human IPF lungs by the Query method and the Overlap method. (**A**) Enrichment z-score summary of 30 cell types in four histopathological region types from spatial proteomics. (**B**) Enrichment *p*-value summary of 30 cell types in four histopathological region types from spatial proteomics. (**C**) Spearman’s correlations between cell type rankings in four histopathological region types from the Query method and the Overlap method.

For the Query method, the first step is to extract up-regulated region-specific proteins by differential analysis. Spatial protein profiling from the Herrera spatial proteomics dataset showed 2835 protein expressions within four histopathological regions: control alveoli, IPF fibroblast foci, IPF fibrosis and IPF alveoli. An unpaired two-tailed Student’s *t*-test (*p* < 10^−2^, calculate effect size with maximum Cohen’s d value) was used to identify up-regulated region-specific DE proteins between the target region types versus all other three non-target types. These protein sets are defined as signatures representing their respective regions (Supplementary Table S19). The average expression levels of genes corresponding to these region-specific proteins were then calculated in each cell type for each region type (Supplementary Table S20), resulting in the enrichment (Figure 3A) and depletion (Supplementary Figure S5A) z-score map for each cell type/region pair.

For the Overlap method, the significance of the overlap items between up-regulated regions-specific proteins and cell type-specific genes was calculated to determine whether their overlap is higher or lower than expected by chance. The resulting association matrix is listed in Supplementary Table S21 and illustrated in Figure 3B (enrichment) and Supplementary Figure S5B (depletion). Moreover, the overlapping up-regulated region-specific proteins and cell type-specific genes in all region/cell type pairs are detailed in Supplementary Tables S22–S25. These signature proteins share common items with previous signature genes in the same histopathological regions (Supplementary Table S9 for gene signatures and Table S22 for protein signatures in control alveoli, Table S11 for gene signatures and Table S25 for protein signatures in IPF alveoli, Table S12 for gene signatures and Table S23 for protein signatures in fibroblast foci), which are highly confident cell type markers abundant in both proteins and mRNAs for respective pathological regions. For instance, LTBP1 (Latent-transforming growth factor beta-binding protein 1) and FN1 (Fibronectin 1) label the myofibroblast cells in the fibroblast foci. These markers can be employed to target specific cell types in select pathological regions for potential cell-based and gene-based therapies. Finally, 30 cell type enrichment z-scores from the Query method and significance *p*-values from the Overlap method in each of the four region types are ranked together, and they display strong positive correlations in corresponding region types (Figure 3C), further confirming the overall integration results.

Both the Query method and the Overlap method indicated that IPF fibroblast foci region is enriched with multiple mesenchymal cell types, including fibroblasts and myofibroblasts, as well as epithelial cells, including basal and KRT5-/KRT17+ epithelium (Figure 3A,B). Control alveoli and IPF fibrosis regions are enriched with various large cell types. Interestingly, IPF alveoli region still shows significant epithelial cell type enrichments compared with control alveoli based on the Query method. However, there are no clear differences in cell type clustering between IPF alveoli and control alveoli based on the Overlap method.

### 3.5. Comparisons of Integration Results from Spatial Transcriptomics and scRNA-Seq Transcriptomics Versus Those from Spatial Proteomics and scRNA-Seq Transcriptomics

**Figure 4:**
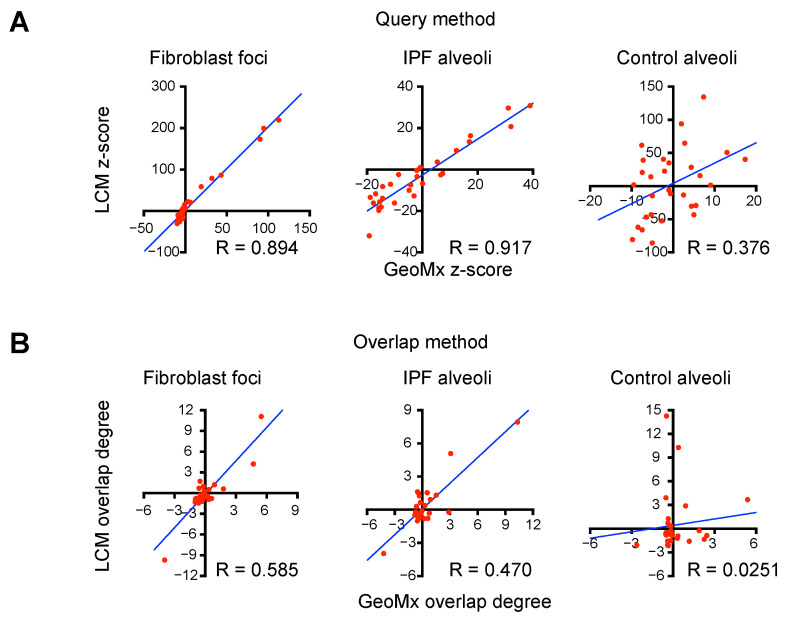
Cell type enrichment comparisons by combining either GeoMx spatial transcriptomics or LCM-directed LC–MS spatial proteomics with scRNA-seq transcriptomics. (**A**) z-score comparisons by the Query method from spatial RNA/cellular RNA integration and spatial protein/cellular RNA integration in 30 cell types in three common region types: IPF fibroblast foci, IPF alveoli and control alveoli regions. (**B**) significance *p*-value comparisons by the Overlap method from spatial RNA/cellular RNA integration and spatial protein/cellular RNA integration in 30 cell types in three common region types: IPF fibroblast foci, IPF alveoli and control alveoli regions. Spearman’s correlations are calculated in each comparison.

So far, cell type enrichments in distinct histopathological region types with the Query method and the Overlap method by combining either GeoMx spatial transcriptomics or LCM-directed LC–MS spatial proteomics with scRNA-seq transcriptomics data. It is natural to ask whether integrations from spatial RNA/cellular RNA and spatial protein/cellular RNA show consistent enrichment results in three common histopathological region types (IPF fibroblast foci, IPF alveoli and control alveoli). For the Query method, we compared z-scores from spatial RNA/cellular RNA and spatial protein/cellular RNA integration in 30 cell types in three common regions (Figure 4A). For the Overlap method, we compared significant *p*-values from spatial RNA/cellular RNA and spatial protein/cellular RNA in 30 cell types in three common regions (Figure 4B). Overall, cell type enrichment rankings from spatial RNA/cellular RNA and spatial protein/cellular RNA show strong positive correlations. The quantitative Query method shows better correlations compared with the qualitative Overlap method (Figure 4A,B). Accordingly, control alveoli show poor cell type enrichment ranking alignment from the Overlap method, likely due to the variations from cross-study region selection. Ideally, GeoMx spatial transcriptomics and LCM-directed LC–MS spatial proteomics should be applied to the same alveolar region from a control donor lung tissue block. However, even though IPF fibroblast foci and IPF alveoli samples come from different studies and are processed with different feature domains (RNA versus protein), their cell type enrichment rankings are strikingly consistent. Together, we adopted two integration methods (Query and Overlap) to integrate spatial multi-omics data with scRNA-seq data to successfully deduce enriched cell types and cell type signatures in histologically defined areas in human IPF lungs based on DE gene or protein features.

## 4. Discussion

IPF lungs exhibit high-level spatial and temporal heterogeneity, characterized by distinct disease-associated niches with spatially discrete cell type compositions during disease progression [20]. Therefore, it is imperative to identify cell types/clusters and signature gene/protein features associated with specific pathological regions, particularly fibroblast foci, to investigate how they drive the fibrogenesis microenvironment. In this study, a stepwise approach was employed to integrate spatial omics with scRNA-seq transcriptomics to investigate the cell type/cluster associations in distinct histopathological regions. First, we evaluated two correlation score-based enrichment methods (Query and Overlap) to integrate GeoMx spatial transcriptomics with scRNA-seq transcriptomics. The overlapping gene features identified by the Overlap method can serve as excellent cell type markers in respective regions. Both methods pinpointed cell type enrichments concordant with previous deconvolution and Xenium-based cell counting methods. Next, we employed in-house LCM-directed LC–MS to profile proteins in histopathological regions from IPF lung tissues. Common DE proteins and enriched pathways were identified, aligning with findings from another published spatial proteomics IPF dataset. Critically, we innovatively expanded the Query and Overlap integration principles to combine LCM-directed LC–MS spatial proteomics with scRNA-seq transcriptomics, which has not been previously reported for IPF. Due to the relatively comprehensive region types with normal control donors and larger sample sizes compared with the in-house dataset, we focus on the published spatial proteomics dataset for this novel integration. This spatial protein/cellular RNA cross-domain integration yielded coherent cell type enrichments comparable to those from spatial RNA/cellular RNA integration, demonstrating the capacity and versatility of the entire approach. This workflow presented in this study can serve as a representative use case for investigating other diseases with spatial heterogeneity.

There will still be a need to confirm spatial transcriptomics findings with spatially resolved proteomics, such as targeted multiplexed immunostaining with high-content imaging or untargeted LCM-directed quantitative LC–MS proteomics since proteins are the final product of mRNA [26]. This is why we combine GeoMx spatial transcriptomics and LCM-directed LC–MS spatial proteomics using differential expression features and enriched pathways as comparison endpoints and cross-validate them with each other before the integration analysis. Spatially resolved single-cell proteomics would be superior to transcriptomics in identifying driver cell types and key protein signatures, particularly ECM components, in pathological regions. However, quantitative untargeted single-cell proteomics is currently unfeasible [26]. Therefore, integrating LCM-directed LC–MS spatial proteomics, which lacks cell type information, with scRNA-seq transcriptomics as an alternative reference is the most relevant stepwise approach. In this study, we successfully reapplied the Query and Overlap enrichment methods evaluated from spatial RNA/cellular RNA integration to combine spatial protein/cellular RNA. To our knowledge, this has not been performed previously, and it results in coherent cell type associations in histopathological regions.

Two correlation score-based enrichment methods (Query and Overlap) were first evaluated to integrate spatial transcriptomics and single-cell transcriptomics data. Both the Query method and the Overlap method assume if a cell type is enriched in a particular region, the cell type and the region should express common DE gene features. As a starting point, up-regulated region-specific DE gene features were extracted from the spatial transcriptomics dataset. Then, the Query method measures the sum expression level of these gene features in distinct cell types from the scRNA-seq transcriptomics data. The Overlap method determines whether region-specific and cell type-specific DE genes overlap more than expected by chance, indicating enrichment. The Query method quantifies gene expression levels in a cell type, while the Overlap method qualitatively counts the overlapping features. In addition, the cell type enrichment z-scores from the Query method are more clustered within large cell types compared with the enrichment significance *p*-values from the Overlap method (comparing Figure 1D and Figure 2C). The correlations between spatial RNA/cellular RNA integration and spatial protein/cellular RNA integration are also stronger using the Query method compared with the Overlap method (Figure 4A,B). Therefore, we propose that the Query method can more reliably detect cell type/region associations compared with the Overlap method in future applications.

Overall, the Query and the Overlap enrichment methods, along with the widely used inference-based deconvolution method are complementary with each other in integrating spatial/cellular transcriptomics. The deconvolution method finds cell type features from scRNA-seq reference data and deduces the proportion of cell types in each ROI from spatial transcriptomics data to obtain average cell type composition within the same pathological region type [25]. Compared with the deconvolution method that deduces cell type abundances in a specific region type, the Query and the Overlap methods emphasize the relative enrichment of the same cell type among distinct histopathological region types. They are relatively less comprehensive, as the deconvolution method needs the entire spatial transcriptomics expression as input, while they only use DE gene features. However, the enrichment methods can integrate spatial proteomics and single-cell transcriptomics in the subsequent analysis, which is a remarkable advantage over the deconvolution method. Both methods rely on identifying unique genes expressed by different cell types, making them highly dependent on cell type annotations from scRNA-seq data references [25,34]. Different cell type/cluster annotations can lead to varying results. In this study, we compared the cell type enrichment ranking results with cell type abundances from two independent deconvolution studies [17,18]. These two orthogonal methods produced consistent cell type-region associations (Supplementary Table S14), confirming each other’s findings.

Both enrichment and deconvolution methods are computation-based omics meta-analysis used to deduce cell type associations in a specific region. Recently, advancements in imaging, barcoding and sequencing technologies have driven the rapid development of spatially resolved single-cell transcriptomics platforms, including Molecular Cartography (Resolve Biosciences), MERSCOPE (Vizgen), Xenium in situ (10x Genomics) and CosMx (NanoString) [43]. These platforms can directly detect predesigned gene panel expression with subcellular resolution while conserving regional information, enabling further investigation of cell-cell interactions through computational biology approaches, which could not be achieved through lower spatial resolution technologies. The current typical Xenium gene panel size is ~400 genes, and CosMx is ~1000 genes [43]. We referred to a recent BioRixiv Xenium paper (customized panel with 343 genes) in human IPF lungs [28] and discovered that the cell type enrichment results from spatial/cellular RNA by integration align with cell type proportion results in the same pathological regions (fibroblast foci and IPF alveoli) from spatially resolved cellular RNA by Xenium (Supplementary Table S14), further corroborating our conclusions. Moreover, third-party independent methods like multiplexed immunofluorescence staining can further validate the appearance of key cell types and signatures identified by either the integrative computation or the Xenium experiment.

Current spatially resolved single-cell transcriptomics platforms, such as Xenium, are limited by targeted gene panel selection, which is not designed for “unbiased” discovery-based efforts to finely define novel cell types or subclusters. Additionally, these approaches are also limited by availability, cost and the precision of cell boundary segmentation [43]. In contrast, the integration approaches utilize spatial omics data like GeoMx and LCM-directed LC–MS with larger untargeted feature domain size. This allows for more comprehensive analysis. scRNA-seq from bulk tissues can also provide better cell type annotation and transcriptome coverage, albeit at the cost of losing regional information. Our integration approach can balance these aspects by combining both data types. However, those abovementioned platforms are advantageous in free offline ROI selection with in situ subcellular resolution [43,44]. Recently, Xenium released the Prime 5K Gene Expression Panels in 2024 May (https://www.10xgenomics.com/support/software/xenium-panel-designer/latest/tutorials/pre-designed-xenium-prime-5k/, accessed on 10 October 2024), and CosMx showcased their Human 6K Discovery Panel in 2024 February (https://NanoString.com/products/cosmx-spatial-molecular-imager/cosmx-rna-assays/human-6k-discovery-panel/, accessed on 10 October 2024). These panels could serve as future data types for spatially resolved single-cell transcriptomics when applied to IPF lungs, despite challenges related to expense, time, and labor work.

Our integration of spatial omics and scRNA-seq transcriptomics reveals spatially discrete distributions and alterations in cell populations associated with IPF pathogenesis. Cell type compositions undergo remarkable reorganization from normal to IPF tissue types, characterized by large cell type clustering. For example, IPF immune infiltrate is uniquely enriched with myeloid and lymphoid cells while IPF alveoli and fibroblast foci regions are depleted, suggesting that these immune cells fail to penetrate the stiffened stroma surrounding the fibroblast foci, similar to the cases observed in some solid fibrotic cancers (e.g., pancreatic tumor) [45,46]. IPF fibroblast foci region is highly enriched with mesenchymal cells like fibroblasts, myofibroblasts and lipofibroblast-like PLIN2+ fibroblasts, as expected for these active fibrosis centers [19,47]. The morphologically normal alveolar regions in IPF lungs exhibit striking enrichments of epithelial cells featuring transitional AT2, basal and KRT5-/KRT17+ epithelial populations linked to impaired regeneration. A recent Visium spatial transcriptomics study in IPF proposed that transitional AT2 cells have diminished capacity for differentiating into regenerative AT1 cells and instead diverge to intermediate, non-regenerative KRT5-/KRT17+ path in IPF lungs [20]. In addition, basal cells act as bronchial stem cells upon lung injury and invade into alveolar epithelium, suggesting progressive loss of alveolar progenitor cells [48]. Distinct fibrotic niches in IPF alveoli are further uncovered by subcellular resolution imaging-based Xenium spatial transcriptomics [28]. In summary, these bioinformatics integrative results suggest early cellular dysfunction and remodeling even in relatively preserved alveolar areas, which is consistent with previous experiment-based reports [20,28]. Future experiment design needs careful spatial and temporal selection of ROIs to investigate early IPF pathogenesis.

Integrative analysis from this study enables the linking of cell types/clusters and gene/protein signatures with histopathological annotations. Using the Overlap method to integrate spatial RNA/cellular RNA and then spatial protein/cellular RNA, we identified overlapping key signature genes/proteins with high confidence. They could mark pathogenic cell subpopulations in specific histopathological regions and represent potential therapeutic targets. For example, LTBP1 and FN1 mRNA and protein expression levels are markedly elevated, specifically in myofibroblasts within fibroblast foci, as defined by the integration approach. LTBP1, or latent transforming growth factor β-binding protein 1, shows increased expression in human IPF lungs, particularly within fibroblast foci. It is involved in the activation of transforming growth factor β (TGF-β), a critical cytokine promoting myofibroblast differentiation and ECM deposition in fibrogenesis [49,50]. Eupatilin, a compound studied for its anti-fibrotic properties, specifically inhibits LTBP1 expression and dismantles latent TGF-β complex to block myofibroblast differentiation [51]. FN1, or fibronectin 1, is a glycoprotein that is significantly upregulated in human IPF lungs in vivo and lung fibroblast culture in vitro upon stimulation with TGF-β, driving fibroblast to myofibroblast differentiation [52,53]. Cell culture and animal models have shown that inhibitors of specific signaling pathways can effectively reduce FN1 levels. Histone acetyltransferase p300 and discoidin domain receptor 1 inhibitors synergistically lower FN1 expression, thus inhibiting fibrosis in both fibroblast culture and bleomycin-induced mouse fibrosis models [54]. Similarly, RNA-binding proteins like human antigen R (HuR) have been demonstrated to stabilize FN1 mRNA and boost FN1 expression. Targeting HuR can disrupt this stabilization, reducing FN1 and other fibrotic markers [53]. These reports suggest LTBP1 and FN1 as crucial targets for therapeutic interventions aimed at attenuating fibrosis in IPF patients. From these comparisons, the bioinformatics analysis shows consistent cellular markers and potential targets with previous experimental reports, increasing the confidence to extend the entire workflow to other disease areas with spatial heterogeneity for broader applications.

There are some limitations to the current study. First, the Eyres GeoMx spatial transcriptomics dataset used is limited to ~1000 genes. Some regions without prominent spatial gene features cannot be integrated with scRNA-seq transcriptomics, such as control blood vessel and IPF adjacent alveoli in this study. The patient sample sizes are also limited. In the future more comprehensive omics datasets with larger omics coverage and sample size could help solve the issue. Second, spatial transcriptomics and spatial proteomics data are from separate studies with different patient cohorts. Therefore, the pathological regions are artificially aligned from spatial transcriptomics and proteomics datasets based on common histopathological annotations. Ideally, both spatial transcriptomics and proteomics slides should come from the same patient tissue block by serial sections with the same histopathological annotation. At present, this type of dataset is not available. Third, the currently used GeoMx spatial transcriptomics and LCM spatial proteomics datasets are still based on relatively large tissue area selections due to sensitivity limitations. These spatial omics approaches are constricted by spatial resolution. Therefore, more in-depth biological insight, e.g., cell-cell interaction investigation, is not feasible. Based on the rapid technological advancements (e.g., CosMx, Visium and Xenium for spatial transcriptomics platform [43], as well as Deep Visual Proteomics and nanoPOT for spatial proteomics platform [26]), tiny regions or niches could be annotated and dissected for spatial proteomics analysis to provide higher cell type specificity. Lastly, the most important sources of proteoform complexity in disease states are alternative splicing and protein post-translational modification (PTM). Some IPF-related proteoform signatures may represent novel pathological cell types/subclusters. Differential analysis on overall protein expression levels has masked the alterations from these splicing isoforms or PTMs. However, the analysis and untargeted quantifications of proteoforms at the intact level are highly challenging and await future technical breakthroughs.

## 5. Conclusions

In summary, we evaluated integrative methods to combine GeoMx spatial transcriptomics with scRNA-seq transcriptomics data to identify coherent diseased cell types and representative gene signatures in distinct pathological regions in human IPF lungs. We then selected the enrichment approach for integrating LCM-directed LC–MS spatial proteomics with scRNA-seq transcriptomics and successfully performed cross-domain spatial multi-omics data integration. Both integration results indicate coherent cell type/region associations in lung fibrosis and highlight the early IPF disease development with discrete cell type composition alternations in morphologically normal adjacent alveoli regions as well as fibroblast foci regions. This provides valuable insights for future mechanistic studies in IPF pathogenesis. The approach can be applied to other disease areas characterized by spatial heterogeneity, such as cancer and autoimmune diseases.

## Figures and Tables

**Table 1:** List of published omics datasets used in the current integration and comparison.

Year	Spatial Data Reference	Spatial Data Access	Feature Domain	Platform	Spatial Method	scRNA-Seq Data Reference	scRNA-Seq Data Access	scRNA-Seq Integration Method	Who Performed the Integration
2022	Eyres [17]	Table S1	RNA	GeoMx DSP	Histopathology directed area selection	Habermann [21]	GSE135893	Query/Overlap/ Deconvolution	This study
2022	Blumhagen [18]	N/A	RNA	GeoMx DSP	Histopathology directed area selection	Adams [22]	GSE136831	Deconvolution	Original paper
2023	Vannan [28]	GSE 250346	RNA	Xenium in situ	Pixel-based whole area, ROI selection offline	N/A	N/A	Direct cell count	Original paper
2022	Herrera [19]	PXD 029341	Protein	LCM-directed LC–MS	Histopathology directed area selection	Habermann [21]	GSE135893	Query/Overlap	This study

## Data Availability

The mass spectrometry proteomics raw files have been deposited to the ProteomeXchange Consortium via the PRIDE partner repository (https://www.ebi.ac.uk/pride/, accessed on 6 January 2025) with the dataset identifier PXD058805 [55].

## Supplementary Materials

The following supporting information can be downloaded at: https://www.mdpi.com/article/10.3390/proteomes13010003/s1, Supplementary Figure S1. The Eyres GeoMx spatial transcriptomics and The Herrera LCM-directed LC–MS spatial proteomics derive consistent feature markers and enriched pathways. (**A**) Total and ECM feature ID coverage summary of GeoMx transcriptome and LCM-directed LC–MS proteome. (**B**) Differential expression of 320 common features between transcriptomics and proteomics with Spearman’s correlations. (**C**-**E**) Comparisons of DE genes and proteins comparing fibroblast foci versus IPF alveoli (**C**), fibroblast foci versus control alveoli (**D**) and IPF alveoli versus control alveoli (**E**) among 320 commonly detected features. Dashed lines represent the FDR equaling 0.05. (**F**) Top 15 GO biological processes from up-regulated DE genes (FC > 1.5 and FDR < 0.05) comparing fibroblast foci and IPF adjacent alveoli ranked by enrichment ratios by ORA from the Eyres transcriptomics dataset. (**G**) Top 15 GO biological processes from up-regulated DE proteins (FC > 1.5 and FDR < 0.05) comparing fibroblast foci and IPF adjacent alveoli ranked by enrichment ratios by ORA from the Herrera proteomics dataset. Supplementary Figure S2. Detailed workflow of the Query (**A**) and Overlap (**B**) methods on data pre-processing and integration steps. Supplementary Figure S3. Cell type depletions in distinct region types integrating GeoMx spatial transcriptomics and scRNA-seq transcriptomics in human IPF lungs by the Query method and the Overlap method. (**A**) Query method: depletion z-score summary of 30 cell types in five histopathological region types from spatial transcriptomics. (**B**) Overlap method: depletion *p*-value summary of 30 cell types in five histopathological region types from spatial transcriptomics. Supplementary Figure S4. LCM-directed LC–MS spatial proteomics identifies protein signatures and enriched pathways in human IPF lungs. (**A**) Optimized proteomics workflow. (**B**) Human IPF lung FFPE specimens were serially sectioned at 5 μm thickness and stained with H&E to annotate fibroblast foci, mature fibrosis and IPF alveoli regions by pathologists. Dashed lines indicate LCM cutting outlines. (**C**) ECM protein IDs summary. (**D**–**F**) Volcano plot showing DE ECM proteins comparing different regions. The horizontal red dashed line indicates a nominal *p*-value of 0.05, and the vertical red dashed lines indicate a 1.5-fold change in protein abundances. (**G**) Venn graphs comparing total and ECM protein IDs between the in-house dataset and the Herrera proteomics dataset. (**H**) DE proteins (|FC| > 1.5 and nominal *p*-value < 0.05) comparing fibroblast foci and IPF alveoli regions from in-house and Herrera data are annotated by gene symbols. The red dashed lines indicate a 1.5-fold change in respective protein abundance. (**I**) Top 15 GO biological processes from up-regulated DE proteins (FC > 1.5 and nominal *p*-value < 0.05) comparing fibroblast foci and IPF alveoli regions ranked by enrichment ratios by ORA from in-house proteomics dataset. Supplementary Figure S5. Cell type depletions in distinct region types integrating LCM-directed LC–MS spatial proteomics and scRNA-seq transcriptomics in human IPF lungs by the Query method and the Overlap method. (**A**) Query method: depletion z-score summary of 30 cell types in four histopathological region types from spatial proteomics. (**B**) Overlap method: depletion significance *p*-value summary of 30 cell types in four histopathological region types from spatial proteomics. Supplementary Table S1. Detailed histological region annotation in the Eyres GeoMx spatial transcriptomics [17] and the Herrera spatial proteomics dataset [19]. Supplementary Table S2. List of over-representative Gene Ontology Biological Process from up-regulated DE genes (Log FC > 0.25, FDR < 0.05) comparing fibroblast foci versus IPF adjacent alveoli regions from the Eyres spatial transcriptomics dataset. Supplementary Table S3. List of over-representative Gene Ontology Biological Process from up-regulated DE proteins (FC > 1.5, FDR < 0.05) comparing fibroblast foci versus IPF adjacent alveoli regions from the Herrera spatial proteomics dataset. Supplementary Table S4. List of up-regulated region-specific genes from the Eyres GeoMx spatial transcriptomics in control alveoli, IPF blood vessel, IPF distant alveoli, IPF fibroblast foci and IPF immune infiltrate regions. Supplementary Table S5. z-score summary of the Query method integrating the Eyres GeoMx spatial transcriptomics and the Habermann scRNA-seq transcriptomics. Supplementary Table S6. List of up-regulated cell type-specific genes from the isolated individual cells in IPF patients from the Habermann scRNA-seq transcriptomics. Supplementary Table S7. List of up-regulated cell type-specific genes from the isolated individual cells in normal control donors from the Habermann scRNA-seq transcriptomics. Supplementary Table S8. significance *p*-value summary (−Log10 (*p*-value)) of the Overlap method integrating the Eyres GeoMx spatial transcriptomics and the Habermann scRNA-seq transcriptomics. Supplementary Table S9. Cell type signature genes of control alveoli derived from the Overlap method. ECM genes are green highlighted. Supplementary Table S10. Cell type signature genes of IPF blood vessel derived from the Overlap method. ECM genes are green highlighted. Supplementary Table S11. Cell type signature genes of IPF distant alveoli derive from the Overlap method. ECM genes are green highlighted. Supplementary Table S12. Cell type signature genes of IPF fibroblast foci derived from the Overlap method. ECM genes are green highlighted. Supplementary Table S13. Cell type signature genes of IPF immune infiltrate derived from the Overlap method. ECM genes are green highlighted. Supplementary Table S14. Comparisons among cell type enrichment rankings from the Query and Overlap methods, cell type proportion estimates from the deconvolution method and cell number proportion ranking from the Xenium measurement. The top 3 cell types in the Eyres deconvolution method are color-highlighted. The same or similar cell type annotations are highlighted with the same color in other methods. Supplementary Table S15. Differential expression analysis of all proteins comparing fibroblast foci versus IPF alveoli regions. Supplementary Table S16. Differential expression analysis of all proteins comparing fibrosis versus IPF alveoli regions. Supplementary Table S17. Differential expression analysis of all proteins comparing fibroblast foci versus fibrosis regions. Supplementary Table S18. List of over-representative Gene Ontology Biological Process from up-regulated DE proteins (FC >1.5, nominal *p*-value < 0.05) comparing fibroblast foci versus IPF alveoli regions. Supplementary Table S19. List of up-regulated region-specific proteins from the Herrera LCM-directed LC–MS spatial proteomics in control alveoli, IPF fibrosis, IPF fibroblast foci and IPF alveoli regions. Supplementary Table S20. z-score summary of the Query method integrating the Herrera LCM-directed LC–MS spatial proteomics and the Habermann scRNA-seq transcriptomics. Supplementary Table S21. significance *p*-value summary (− Log10 (*p*-value)) of the Overlap method integrating the Herrera LCM-directed LC–MS spatial proteomics and the Habermann scRNA-seq transcriptomics. Supplementary Table S22. Cell type signature proteins of control alveoli region derived from the Overlap method. ECM proteins are green highlighted. Overlapping signature proteins of control alveoli region from spatial proteomics with signature genes of control alveoli region from spatial transcriptomics in Supplementary Table S9 are red highlighted. Supplementary Table S23. Cell type signature proteins of IPF fibroblast foci region derived from the Overlap method. ECM proteins are green highlighted. Overlapping signature proteins of IPF fibroblast foci region from spatial proteomics with signature genes of IPF fibroblast foci region from spatial transcriptomics in Supplementary Table S12 are red highlighted. Supplementary Table S24. Cell type signature proteins of IPF fibrosis region derived from the Overlap method. ECM proteins are green highlighted. Supplementary Table S25. Cell type signature proteins of IPF alveoli region derived from the Overlap method. ECM proteins are green highlighted. Overlapping signature proteins of IPF alveoli region from spatial proteomics with signature genes of IPF alveoli region from spatial transcriptomics in Supplementary Table S11 are red highlighted.
