# Supplementary material for: Novel Integration of Spatial and Single-Cell Omics Data Sets Enables Deeper Insights into IPF Pathogenesis
Source: Proteomes. 2025 Jan 13;13(1):3. doi: 10.3390/proteomes13010003 (PMC11755616; doi:10.3390/proteomes13010003)
Supplement: Supplementary file 1 [file proteomes-13-00003-s001.zip › Supplementary figures.pdf]

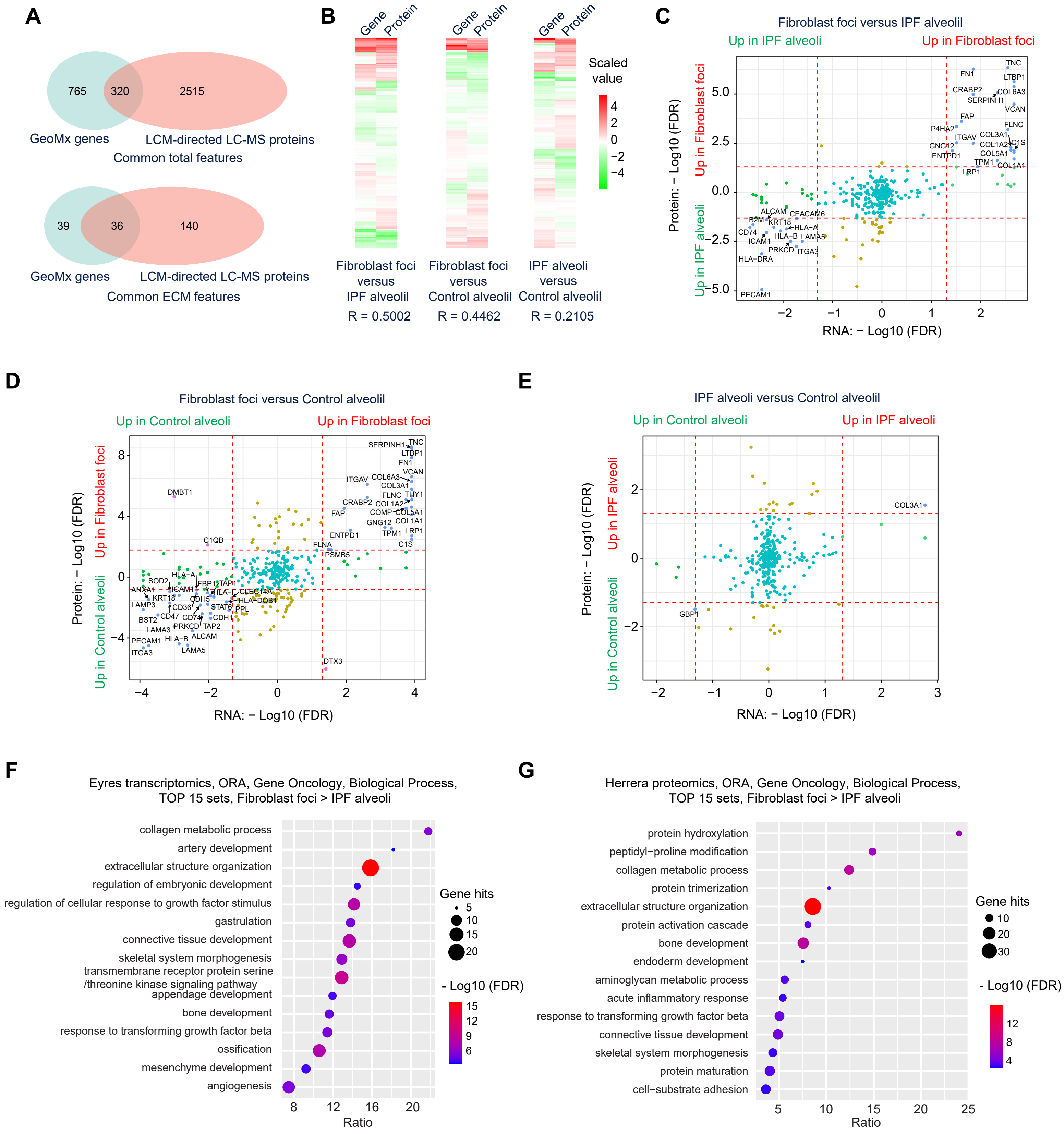

A
765
320
2515
GeoMx genes
LCM-directed LC-MS proteins
Common total features
39
36
140
GeoMx genes
LCM-directed LC-MS proteins
Common ECM features
B
Gene
Protein
Scaled value
Fibroblast foci versus IPF alveolil
R = 0.5002
Fibroblast foci versus Control alveolil
R = 0.4462
IPF alveoli versus Control alveolil
R = 0.2105
C
Fibroblast foci versus IPF alveolil
Up in IPF alveoli
Up in Fibroblast foci
Protein: − Log10 (FDR)
RNA: − Log10 (FDR)
D
Fibroblast foci versus Control alveolil
Up in Control alveoli
Up in Fibroblast foci
Protein: − Log10 (FDR)
RNA: − Log10 (FDR)
E
IPF alveoli versus Control alveolil
Up in Control alveoli
Up in IPF alveoli
Protein: − Log10 (FDR)
RNA: − Log10 (FDR)
F
Eyres transcriptomics, ORA, Gene Oncology, Biological Process, TOP 15 sets, Fibroblast foci > IPF alveoli
collagen metabolic process
artery development
extracellular structure organization
regulation of embryonic development
regulation of cellular response to growth factor stimulus
gastrulation
connective tissue development
skeletal system morphogenesis
transmembrane receptor protein serine /threonine kinase signaling pathway
appendage development
bone development
response to transforming growth factor beta
ossification
mesenchyme development
angiogenesis
Ratio
Gene hits
- Log10 (FDR)
G
Herrera proteomics, ORA, Gene Oncology, Biological Process, TOP 15 sets, Fibroblast foci > IPF alveoli
protein hydroxylation
peptidyl−proline modification
collagen metabolic process
protein trimerization
extracellular structure organization
protein activation cascade
bone development
endoderm development
aminoglycan metabolic process
acute inflammatory response
response to transforming growth factor beta
connective tissue development
skeletal system morphogenesis
protein maturation
cell−substrate adhesion
Ratio
Gene hits
- Log10 (FDR)

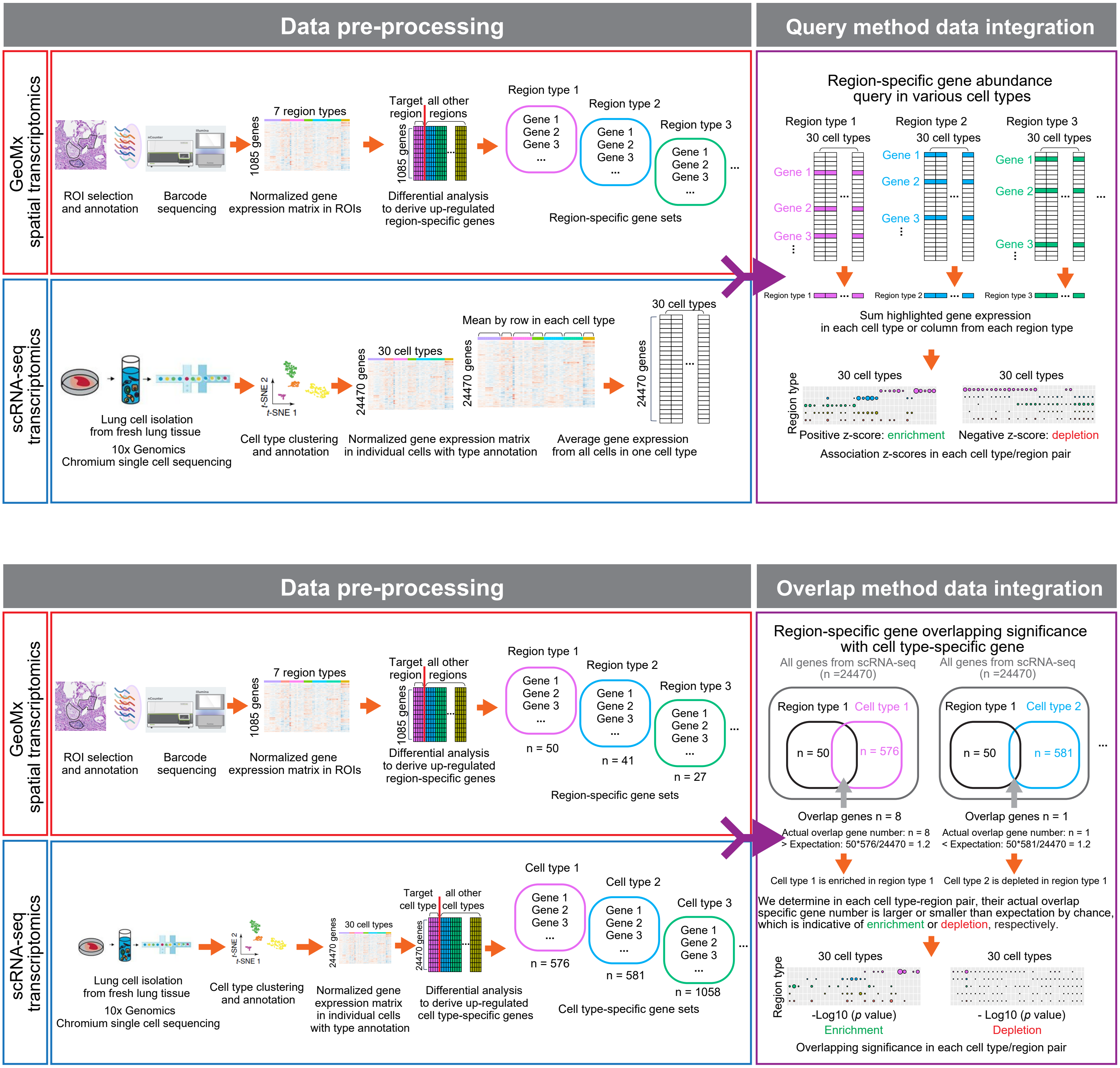
Data pre-processing
Query method data integration
GeoMx spatial transcriptomics
ROI selection and annotation
Barcode sequencing
7 region types
1085 genes
Normalized gene expression matrix in ROIs
Target region
all other regions
Differential analysis to derive up-regulated region-specific genes
Region type 1
Region type 2
Region type 3
Gene 1
Gene 2
Gene 3
Region-specific gene sets
scRNA-seq transcriptomics
Lung cell isolation from fresh lung tissue
10x Genomics Chromium single cell sequencing
t-SNE 1
t-SNE 2
Cell type clustering and annotation
30 cell types
24470 genes
Normalized gene expression matrix in individual cells with type annotation
Mean by row in each cell type
Average gene expression from all cells in one cell type
Region-specific gene abundance query in various cell types
Sum highlighted gene expression in each cell type or column from each region type
Region type
Positive z-score: enrichment
Negative z-score: depletion
Association z-scores in each cell type/region pair
Data pre-processing
Overlap method data integration
n = 50
n = 41
n = 27
Target cell type
all other cell types
Differential analysis to derive up-regulated cell type-specific genes
Cell type 1
Cell type 2
Cell type 3
n = 576
n = 581
n = 1058
Cell type-specific gene sets
Region-specific gene overlapping significance with cell type-specific gene
All genes from scRNA-seq (n =24470)
Overlap genes n = 8
Actual overlap gene number: n = 8
> Expectation: 50*576/24470 = 1.2
Overlap genes n = 1
Actual overlap gene number: n = 1
< Expectation: 50*581/24470 = 1.2
Cell type 1 is enriched in region type 1
Cell type 2 is depleted in region type 1
We determine in each cell type-region pair, their actual overlap specific gene number is larger or smaller than expectation by chance, which is indicative of enrichment or depletion, respectively.
-Log10 (p value)
Enrichment
- Log10 (p value)
Depletion
Overlapping significance in each cell type/region pair

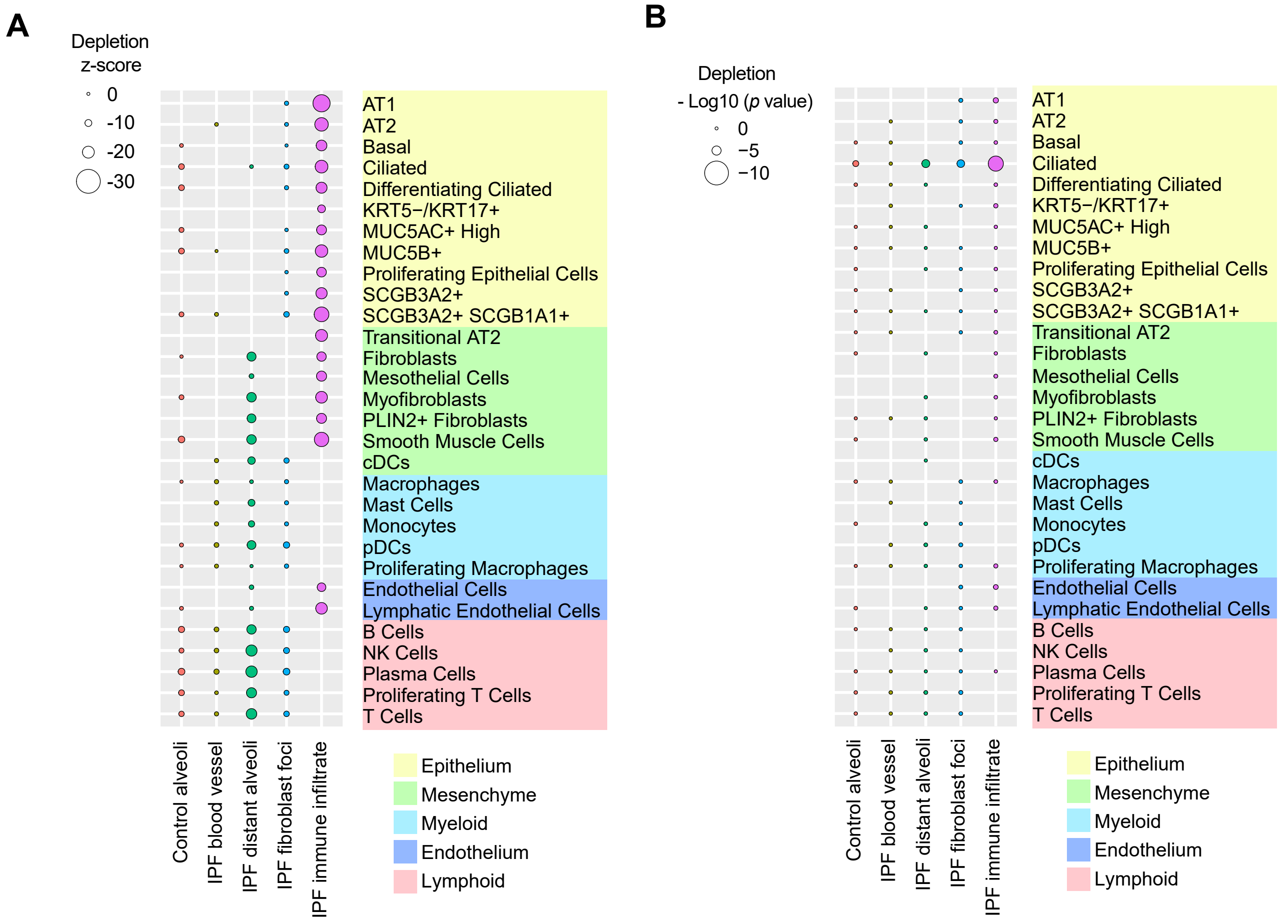
A
Depletion z-score
0
-10
-20
-30
AT1
AT2
Basal
Ciliated
Differentiating Ciliated
KRT5−/KRT17+
MUC5AC+ High
MUC5B+
Proliferating Epithelial Cells
SCGB3A2+
SCGB3A2+ SCGB1A1+
Transitional AT2
Fibroblasts
Mesothelial Cells
Myofibroblasts
PLIN2+ Fibroblasts
Smooth Muscle Cells
cDCs
Macrophages
Mast Cells
Monocytes
pDCs
Proliferating Macrophages
Endothelial Cells
Lymphatic Endothelial Cells
B Cells
NK Cells
Plasma Cells
Proliferating T Cells
T Cells
Control alveoli
IPF blood vessel
IPF distant alveoli
IPF fibroblast foci
IPF immune infiltrate
Epithelium
Mesenchyme
Myeloid
Endothelium
Lymphoid
B
Depletion
- Log10 (p value)
0
−5
−10
AT1
AT2
Basal
Ciliated
Differentiating Ciliated
KRT5−/KRT17+
MUC5AC+ High
MUC5B+
Proliferating Epithelial Cells
SCGB3A2+
SCGB3A2+ SCGB1A1+
Transitional AT2
Fibroblasts
Mesothelial Cells
Myofibroblasts
PLIN2+ Fibroblasts
Smooth Muscle Cells
cDCs
Macrophages
Mast Cells
Monocytes
pDCs
Proliferating Macrophages
Endothelial Cells
Lymphatic Endothelial Cells
B Cells
NK Cells
Plasma Cells
Proliferating T Cells
T Cells
Control alveoli
IPF blood vessel
IPF distant alveoli
IPF fibroblast foci
IPF immune infiltrate
Epithelium
Mesenchyme
Myeloid
Endothelium
Lymphoid

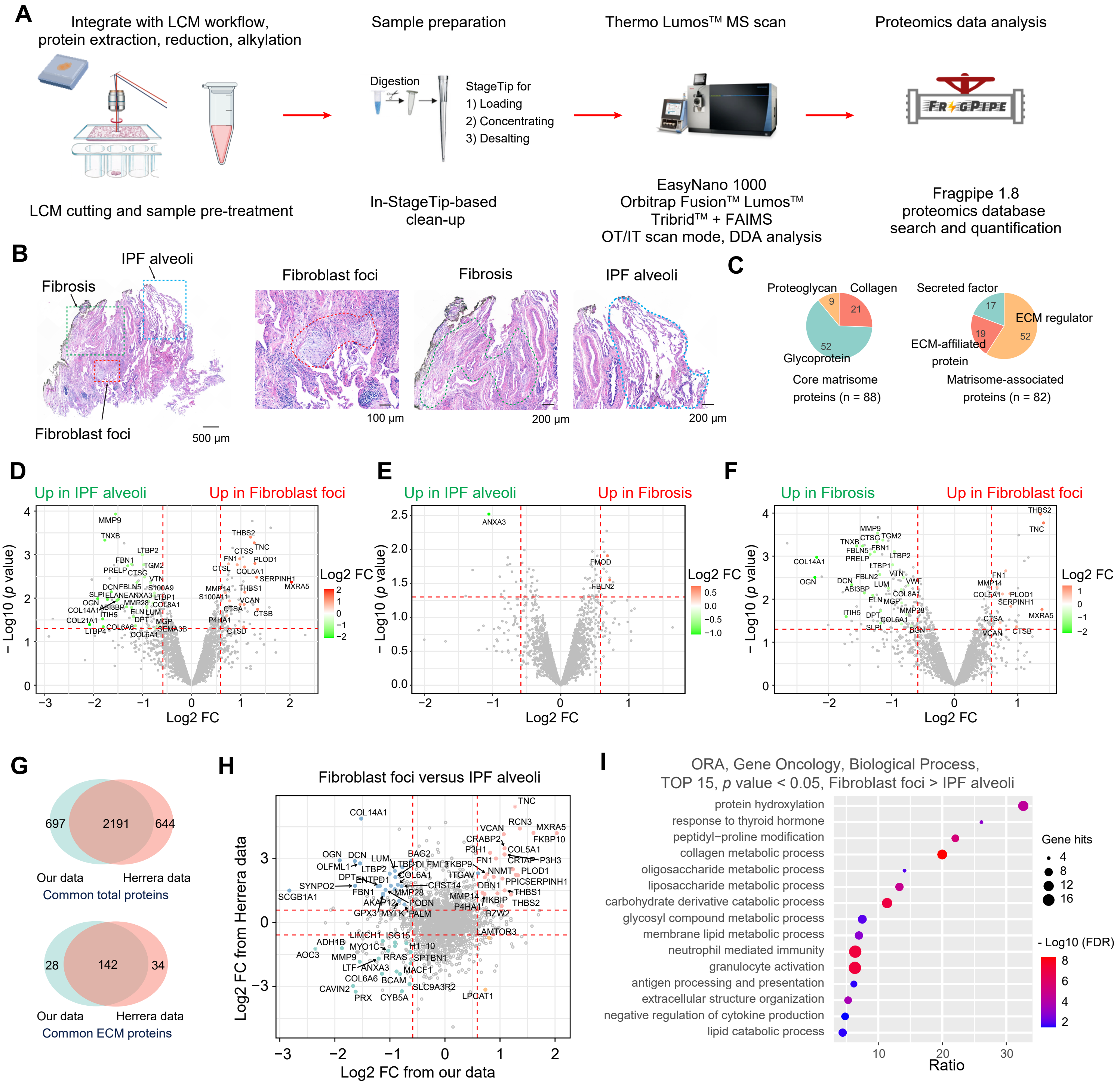
A
Integrate with LCM workflow, protein extraction, reduction, alkylation
LCM cutting and sample pre-treatment
Sample preparation
Digestion
StageTip for
1) Loading
2) Concentrating
3) Desalting
In-StageTip-based clean-up
Thermo Lumos™ MS scan
EasyNano 1000
Orbitrap Fusion™ Lumos™
Tribrid™ + FAIMS
OT/IT scan mode, DDA analysis
Proteomics data analysis
FRAGPIPE
Fragpipe 1.8
proteomics database search and quantification
B
IPF alveoli
Fibrosis
Fibroblast foci
500 µm
100 µm
200 µm
200 µm
C
Proteoglycan
Collagen
Glycoprotein
Core matrisome proteins (n = 88)
Secreted factor
ECM regulator
ECM-affiliated protein
Matrisome-associated proteins (n = 82)
D
Up in IPF alveoli
Up in Fibroblast foci
E
Up in IPF alveoli
Up in Fibrosis
F
Up in Fibrosis
Up in Fibroblast foci
− Log10 (p value)
Log2 FC
G
697
2191
644
Our data
Herrera data
Common total proteins
28
142
34
Our data
Herrera data
Common ECM proteins
H
Fibroblast foci versus IPF alveoli
Log2 FC from Herrera data
Log2 FC from our data
I
ORA, Gene Oncology, Biological Process,
TOP 15, p value < 0.05, Fibroblast foci > IPF alveoli
protein hydroxylation
response to thyroid hormone
peptidyl−proline modification
collagen metabolic process
oligosaccharide metabolic process
liposaccharide metabolic process
carbohydrate derivative catabolic process
glycosyl compound metabolic process
membrane lipid metabolic process
neutrophil mediated immunity
granulocyte activation
antigen processing and presentation
extracellular structure organization
negative regulation of cytokine production
lipid catabolic process
Gene hits
- Log10 (FDR)
Ratio

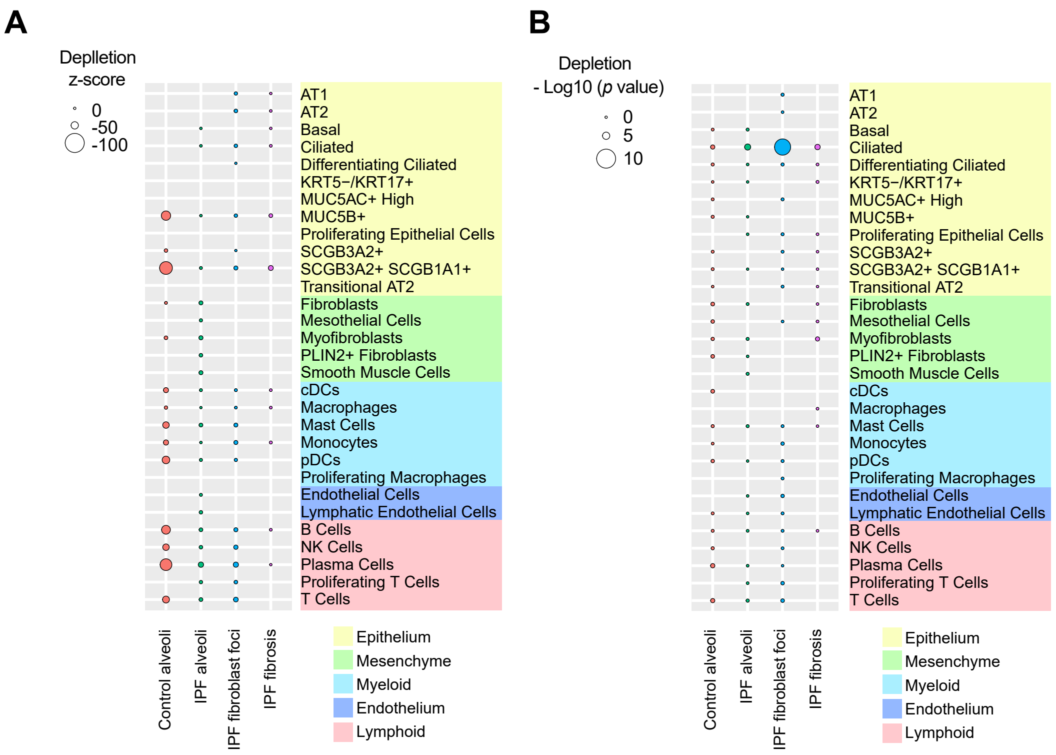
A
Depletion
z-score
0
-50
-100
AT1
AT2
Basal
Ciliated
Differentiating Ciliated
KRT5−/KRT17+
MUC5AC+ High
MUC5B+
Proliferating Epithelial Cells
SCGB3A2+
SCGB3A2+ SCGB1A1+
Transitional AT2
Fibroblasts
Mesothelial Cells
Myofibroblasts
PLIN2+ Fibroblasts
Smooth Muscle Cells
cDCs
Macrophages
Mast Cells
Monocytes
pDCs
Proliferating Macrophages
Endothelial Cells
Lymphatic Endothelial Cells
B Cells
NK Cells
Plasma Cells
Proliferating T Cells
T Cells
Control alveoli
IPF alveoli
IPF fibroblast foci
IPF fibrosis
Epithelium
Mesenchyme
Myeloid
Endothelium
Lymphoid
B
Depletion
- Log10 (p value)
0
5
10
